# Key role of the REC lobe during CRISPR–Cas9 activation by ‘sensing’, ‘regulating’, and ‘locking’ the catalytic HNH domain

**DOI:** 10.1017/S0033583518000070

**Published:** 2018-08-03

**Authors:** Giulia Palermo, Janice S. Chen, Clarisse G. Ricci, Ivan Rivalta, Martin Jinek, Victor S. Batista, Jennifer A. Doudna, J. Andrew McCammon

**Affiliations:** 1 Department of Bioengineering, University of California, Riverside, CA 92507; 2 Department of Molecular and Cell Biology, University of California, Berkeley, Berkeley, CA 94720, USA; 3 Department of Chemistry and Biochemistry, University of California, San Diego, La Jolla, CA 92093, USA; 4 Department of Pharmacology, University of California, San Diego, La Jolla, CA 92093, USA; 5 Université de Lyon, École Normale Supérieure (ENS) de Lyon, CNRS, Lyon 1, France; 6 Department of Biochemistry, University of Zürich, Winterthurerstrasse 190, CH-8057 Zürich, Switzerland; 7 Department of Chemistry, Yale University, P.O. Box 208107, New Haven, CT 06520-8107, USA; 8 Department of Chemistry, University of California, Berkeley, Berkeley, CA 94720, USA; 9 Howard Hughes Medical Institute, University of California, Berkeley, Berkeley, CA 94720, USA; 10 Physical Biosciences Division, Lawrence Berkeley National Laboratory, University of California, Berkeley, Berkeley, CA 94720, USA; 11 National Biomedical Computation Resource, University of California, San Diego, La Jolla, CA 92093, USA; 12 San Diego Supercomputer Center, University of California, San Diego, La Jolla, CA 92093, USA

**Keywords:** CRISPR–Cas9, genome editing, molecular dynamics, protein/nucleic acid interactions

## Abstract

Understanding the conformational dynamics of CRISPR (clustered regularly interspaced short palindromic repeat)–Cas9 is of the utmost importance for improving its genome editing capability. Here, molecular dynamics simulations performed using Anton-2 – a specialized supercomputer capturing micro-to-millisecond biophysical events in real time and at atomic-level resolution – reveal the activation process of the endonuclease Cas9 toward DNA cleavage. Over the unbiased simulation, we observe that the spontaneous approach of the catalytic domain HNH to the DNA cleavage site is accompanied by a remarkable structural remodeling of the recognition (REC) lobe, which exerts a key role for DNA cleavage. Specifically, the significant conformational changes and the collective conformational dynamics of the REC lobe indicate a mechanism by which the REC1–3 regions ‘sense’ nucleic acids, ‘regulate’ the HNH conformational transition, and ultimately ‘lock’ the HNH domain at the cleavage site, contributing to its catalytic competence. By integrating additional independent simulations and existing experimental data, we provide a solid validation of the activated HNH conformation, which had been so far poorly characterized, and we deliver a comprehensive understanding of the role of REC1–3 in the activation process. Considering the importance of the REC lobe in the specificity of Cas9, this study poses the basis for fully understanding how the REC components control the cleavage of off-target sequences, laying the foundation for future engineering efforts toward improved genome editing.

## Introduction

CRISPR (clustered regularly interspaced short palindromic repeat)–Cas9 is a bacterial adaptive immune system, which has revolutionized life sciences through the introduction of a facile genome editing technology (Doudna and Charpentier, 2014; Jinek *et al*., 2012). In this system, the endonuclease Cas9 can be programmed with single-guide RNAs to site-specifically recognize and cleave any DNA sequence bearing a protospacer adjacent motif (PAM) sequence, which serves as a key recognition element across the genome. This enables genetic engineering of biological systems with unprecedented efficiency, resulting in transformative applications in the life sciences, including the fields of medicine and biotechnology.

**Fig. 1.**
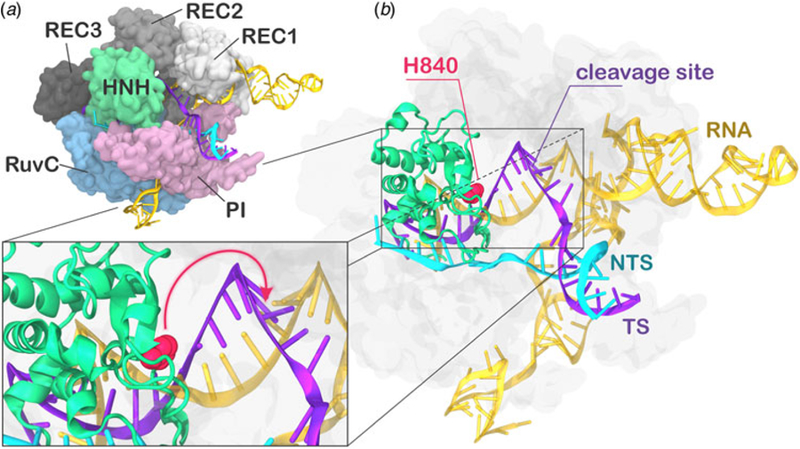
(a) X-ray structure of the *Streptococcus pyogenes* CRISPR-Cas9 system (5F9R.pdb) in the pre-activated state (Jiang *et al*., 2016). Cas9 is shown in molecular surface, highlighting protein domains in different colors. The RNA (orange), the target DNA (TS, magenta), and non-target DNA (NTS, cyan) strands are shown as ribbons. (b) The catalytic residue H840 (magenta) of the HNH domain is located ~19 Å from the cleavage site on the TS. A close-up view of the active site shows the additional conformational change needed to attain the formation of an activated state (shown using an arrow).

During CRISPR–Cas9 activation, the DNA binds Cas9 by matching the guide RNA with one strand (the target strand, TS) and forming an RNA:DNA hybrid, while the non-target strand (NTS) is displaced. Two nuclease domains, HNH and RuvC, catalyze the cleavage of the TS and NTS, respectively. The Cas9 protein comprises a recognition (REC) lobe, which mediates the nucleic acid binding through three recognition domains (REC1–3), and a nuclease lobe including the RuvC and HNH catalytic cores (Fig. 1). At the protein C-terminus, a domain structurally similar to type II topoisomerase constitutes the PAM interacting (PI) region (Jiang and Doudna, 2017; Nishimasu and Nureki, 2017). Structural and biophysical studies have revealed that relevant conformational changes occur upon binding of the nucleic acids (Chen and Doudna, 2017). Specifically, RNA binding primes the protein for subsequent DNA binding (Jiang *et al*., 2015), while the dynamics of the HNH exert conformational control over Cas9 nuclease activity (Sternberg *et al*., 2015). Upon DNA binding, the HNH domain undergoes a structural transition from an inactivated state, in which the catalysis is hampered, to an activated state prone for the cleavage of the TS. While the inactivated state has been well characterized via X-ray crystallography (Anders *et al*., 2014; Nishimasu *et al*., 2014), high-resolution data for the activated state are missing. Indeed, the most complete X-ray structure of Cas9 from *Streptococcus pyogenes* in complex with the nucleic acids captured a pre-activated state of the system, with the HNH catalytic H840 located ~19.4 Å from the cleavage site on the TS (Fig. 1b) (Jiang *et al*., 2016). Clearly, the activation of the system toward catalysis requires a further conformational change of the HNH domain (Fig. 1b, lower panel). Very recently, a novel cryo-EM structure has been reported at a 5.2 Å resolution (EMD-8236) (Huai *et al*., 2017), in which the HNH domain approaches REC1. The difficulty in experimentally capturing the active conformation of CRISPR–Cas9 and the associated conformational transitions reflects the ‘striking flexibility’ of the protein, as arising by the interplay with the nucleic acids, during recognition, association, and cleavage (Palermo *et al*., 2016; Sternberg *et al*., 2015). As a support to the structural characterizations, extensive experimental efforts, including Forster Resonance Energy Transfer (FRET) and structural comparisons with homologous systems, identified the conformational requirements of a catalytically competent Cas9 (Chen and Doudna, 2017). In an initial study, Sternberg *et al*. used bulk FRET to reveal that the conformational dynamics of the HNH domain controls DNA cleavage (Sternberg *et al*., 2015). Subsequently, single-molecule FRET (smFRET) experiments characterized the conformational features of the activated HNH docked at the cleavage site (Dagdas *et al*., 2017), and also revealed that the high flexibility of the REC lobe facilitates the activation of the HNH domain (Chen *et al*., 2017; Osuka *et al*., 2018). Moreover, computational studies have contributed in understanding the conformational dynamics of HNH from the apo form of Cas9 up to the DNA-bound state (Huai *et al*., 2017; Palermo *et al*., 2017a, 2017b; Zuo and Liu, 2017). However, the detailed conformational rearrangements leading up to the catalytically active Cas9 protein have not been clarified. Specifically, it is unclear how HNH would dock at the cleavage site and, importantly, how the REC domains would facilitate this process mechanistically. Understanding the role of the REC lobe in the activation of Cas9 is of key importance for improving the system toward controlled functionality. Indeed, mutations within the REC lobe have been shown to reduce off-target cleavage events in newly evolved CRISPR–Cas9 systems (Casini *et al*., 2018; Chen *et al*., 2017; Kleinstiver *et al*., 2016).

Here, multi-microsecond length molecular dynamics (MD) simulations reveal the activation process in ~16 µs of continuous simulation. Remarkably, the active conformation reached via MD simulations matches the structural and conformational transitions indicated by smFRET, while also being in accord with the newly available cryo-EM data. We show that the transition of the HNH domain depends on the structural remodeling of the REC domains, and is driven by favorable interactions with the REC lobe that form ‘*on the fly*’ during MD, eventually leading to the stable docking of HNH at the cleavage site. The observed conformational changes of the REC components pinpoint on an atomic scale how the recognition domains REC1–3 ‘sense’ nucleic acids, ‘regulate’ the HNH conformational change, and ultimately ‘lock’ HNH at the cleavage site, contributing to its catalytic activation. Finally, tight coupling between the REC lobe and the HNH domain is observed upon activation, ultimately ensuring catalytic competence (Chen *et al*., 2017; Dagdas *et al*., 2017).

## Results

### Conformational transition of the HNH domain

Several studies have been conducted to understand the kinetics of the HNH conformational change, revealing a slow overall conformational transition (i.e. from the inactivated to activated state over milliseconds to seconds) (Raper *et al*., 2018; Shibata *et al*., 2017; Singh *et al*., 2016). However, the final conformational adjustment of the pre-activated crystal structure (Fig. 1) could rapidly occur (i.e. within micro-to-milliseconds), leading to an activated Cas9 (Jiang *et al*., 2016). Due to the high flexibility of the HNH domain (Sternberg *et al*., 2015), the precise determination of its conformational change and the associated kinetics has been limited. In order to test the hypothesis of a fast sub-millisecond conformational change, in our previous study, we have carried out MD simulations of the pre-activated CRISPR–Cas9 (5F9R.pdb) (Jiang *et al*., 2016) in an enhanced sampling regime (Palermo *et al*., 2017a). We employed a Gaussian-accelerated MD (GaMD) approach, which can access conformational states of proteins and nucleic acids over milliseconds (and in some cases beyond) by running much shorter simulations (i.e. of hundreds of nanoseconds) (Miao and McCammon, 2016a, 2018; Miao *et al*., 2015). During ~400 ns of GaMD, the catalytic H840 approached the scissile phosphate on the TS (i.e. phosphate −3) at a distance of ~15.0 Å, from its original location at ~19.4 Å (Fig. S1). Although the catalytic domain remained beyond the range required for catalysis, these simulations have highlighted the tendency for a fast conformational change of HNH in the late step of activation, in agreement with experiments (Dagdas *et al*., 2017; Shibata *et al*., 2017; Sternberg *et al*., 2015). Encouraged by these outcomes, here we performed continuous simulations with the aim of capturing the activation process of the HNH domain in real time and on an atomic scale. Noteworthy, recent studies have employed enhanced sampling methods to access kinetic information (Stelzl and Hummer, 2017), while the efficiency of accelerated MD methodologies in exploring the conformational space has been used in conjunction with Markov models to construct solid kinetic models (Paul *et al*., 2017). However, while enhanced sampling MD describes well thermodynamic properties and conformational ensembles, it does not directly provide kinetic information, the latter is however preserved via unbiased simulations (Abrams and Bussi, 2014; Miao and McCammon, 2016b).

**Fig. 2.**
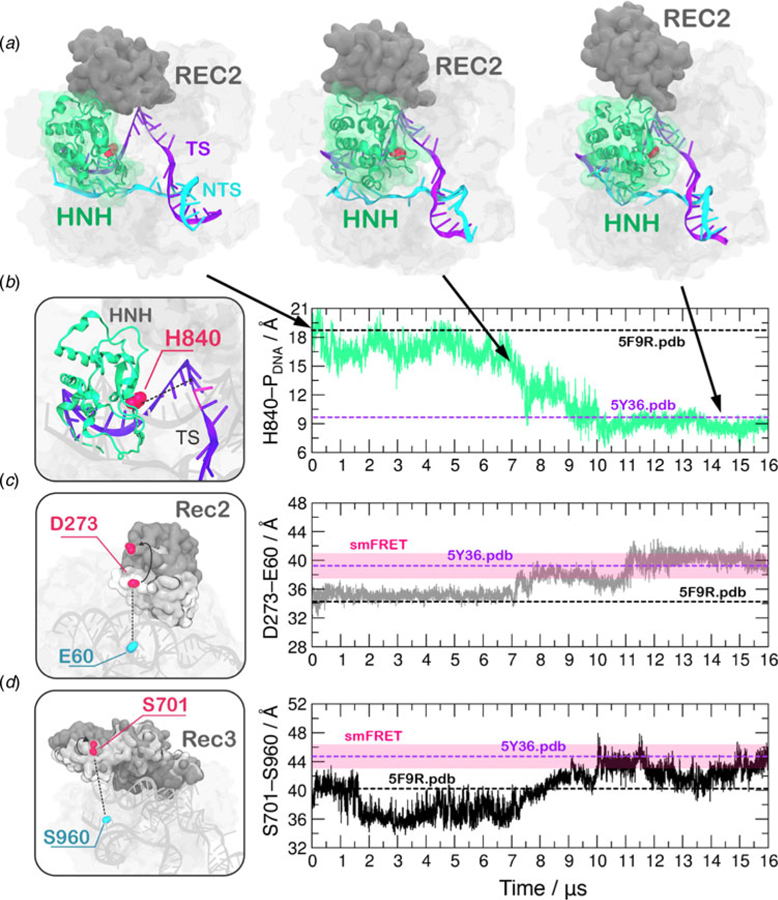
(a) Conformational change of the HNH domain and structural adaptation of REC2 during ~16 µs of continuous MD simulations. (b–d) Time evolution along MD of: (b) the distance between H840 *Cα* and the scissile phosphate on the target DNA strand (phosphate at position −3), indicating the approach of the HNH domain at the cleavage site; (c) the distance between *Cα* atoms of E60 and D273 indicating the outward translation of REC2; (d) the distance between *Cα* atoms of S960 and S701 indicating the conformational change of REC3. Horizontal bars are used to indicate the value of the three distances in the X-ray structure of the pre-activated state (5F9R.pdb, starting configuration for MD) and in the model structure obtained via cryo-EM fitting of the EMD-8236 map (5Y36.pdb) (Huai *et al*., 2017; Jiang *et al*., 2016). Transparent boxes are used to indicate the range assumed by the distances in single molecule Förster Resonance Energy Transfer (smFRET) experiments (Chen *et al*., 2017; Dagdas *et al*., 2017).

In order to obtain a continuous MD trajectory encompassing the microsecond time scale, we carried out MD on a specialized supercomputer – Anton-2 – that enables for micro-to-millisecond length simulations (Shaw *et al*., 2014). CRISPR–Cas9 was simulated for ~16 µs revealing that the HNH domain approaches the cleavage site on the NTS after ~7 µs of MD and stably reaches a catalytically competent state after ~10 µs of MD (Fig. 2a–b).During the conformational transition, the distance between the *Cα* atom of the HNH catalytic residue (H840) and the scissile phosphate on the target DNA strand (i.e. the phosphate atom at position −3) shows a gradual decrease starting at ~7 µs, stabilizing at a distance of ~8 Å from the cleavage site after ~10 µs of MD (Fig. 2b, Fig. S2B). Remarkably, upon ~7 µs of unbiased MD, H840 approached the scissile phosphate at ~15.0 Å, reaching the configuration previously observed via GaMD simulations (Fig. S1). This result further establishes the ability of GaMD to capture long time scale events. However, longer GaMD trajectories might be required to access the complete conformational transition. In the final conformation, the catalytic H840 *Cα* is located ~8 Å from the cleavage site, while the imidazole side chain (and the reactive nitrogen) locates at a distance of ~5–6 Å from the cleavage site, priming the HNH domain for the hydrolysis of the TS (Fig. S3) (Jinek *et al*., 2014). This configuration of the HNH domain agrees well with smFRET experiments (Raper *et al*., 2018; Sternberg *et al*., 2015). Indeed, the S867– S355 distance, previously used to characterize the activated state of the HNH domain, reaches the experimental value of ~21 Å (Fig. S4). In the course of the simulation, we detect significant conformational plasticity of the REC lobe, in agreement with previous characterizations, performed using smFRET experiments (Chen *et al*., 2017; Dagdas *et al*., 2017). Figure 2c–d reports the time evolution during MD of the E60–D273 and S960–S701 distances, which have been used in smFRET to distinguish the con-formational states adopted by the REC2 and REC3 domains, respectively (Chen *et al*., 2017; Dagdas *et al*., 2017). Here, we observe a conformational change of REC2 (Fig. 2a–c). After ~7 µs, simultaneously with the initiation of the conformational rearrangement of HNH, REC2 starts an outward transition that, upon ~11 µs of MD, results in an overall translation by ~8 Å relative to the starting position (i.e. the E60–D273 distance reaches ~40 Å from the initial 32.6 of the X-ray structure 5F9R.pdb). REC3 also shows significant conformational transitions (Fig. 2d). Indeed, the S960–S701 distance broadly fluctuates but overall increases by ~3–3.5 Å (from the initial value of 40.3 of the X-ray structure 5F9R.pdb), resulting in the opening of the groove hosting the RNA:DNA hybrid. Remarkably, in the final configuration the E60–D273 and S960–S701 distances reach values that well agree with the smFRET ranges (Figure 2c–d) (Chen *et al*., 2017; Dagdas *et al*., 2017). Interestingly, the transitions of HNH and REC2 appear to be concerted, as it starts for both domains upon ~7 µs of simulation (Fig. 2b–c). However, HNH reaches an active conformation by 10 µs, while REC2 fully adopts the conformational transition at ~11 µs. Together, these observations suggest that the conformational changes of the recognition lobe assist the conformational activation of the HNH domain. Specifically, we observe an opening of REC3 and the mutual conformational adaptation of REC2 and HNH, whereby HNH approaches the cleavage site on the TS and REC2 moves apart with an outward translation of ~8 Å, enabling the active site to access the cleavage site.

A recent cryo-EM structure, solved at a 5.2 Å resolution (EMD-8236), shows that the HNH domain is closer to the recognition lobe than in the pre-cleavage state crystal structure (Huai *et al*., 2017). The final configuration obtained from MD is in good agreement with the all-atom model fitted in the EMD-8236 EM map (5Y36.pdb, Fig. 2b–d). In detail, in the 5Y36.pdb, the E60–D273 and S960–S701 distances reach values of 38.4 and 42.6 Å, respectively. MD simulations access these values by ~11 µs (for the E60–D273 distance, Fig. 2c) and ~10 µs (for the S960–S701 distance, Fig. 2d). In the fitted structure, the H840 *Cα* atom remains at a distance of 10.1 Å from the scissile phosphate (Fig. 2b), which is beyond the range required for catalysis (Fig. S3). In summary, the continuous MD simulations over-all access the conformation obtained via cryo-EM, and further explore the conformational space. In this respect, it is worth noting given its limited resolution (5.2 Å), the available EMD-8236 structure can in fact represent multiple conformational states that could not be resolved (Nogales, 2016). As such, the fluctuations captured by the extensive MD simulations can be considered representative of the conformational landscape surrounding the EMD-8236 structure.

**Fig. 3.**
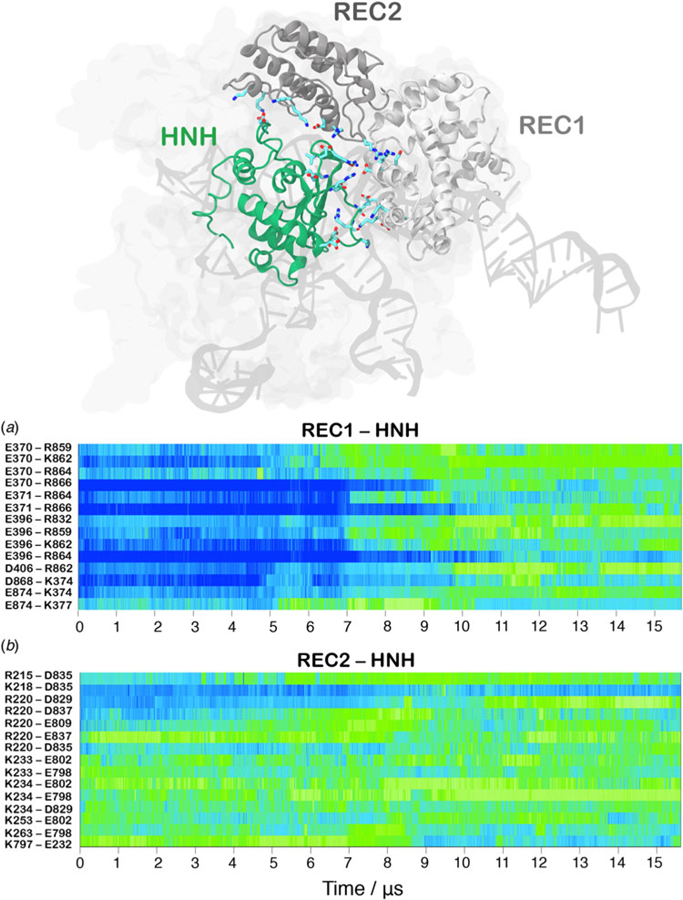
Time evolution along ~16 µs of continuous MD simulations of the CRISPR-Cas9 system of the salt-bridge interactions established between the HNH and REC1 domains (a) and between the HNH and REC2 domains (b). Salt bridges are computed as distance between the center of mass of the oxygen atoms in the acidic side chain and center of mass of the nitrogen atoms in the basic side chain. The scale on the right shows the change in strength of salt-bridge interactions: from weak (blue) to strong (green). The most important ionic interactions established by HNH and the REC1/REC2 regions are also shown on the three-dimensional structure of the activated CRISPR–Cas9.

In order to track the large-scale collective motions of the REC lobe during the long time scale dynamics, we performed Principal Component Analysis (PCA) (Amadei *et al*., 1993). This analysis captures how the protein domains move with respect to each other, highlighting conformational changes that are difficult to observe by visual inspection of the MD trajectory. As a result, PCA confirms large amplitude motions for HNH and the REC2–3 domains and the concerted nature of the conformational changes, while also revealing a conformational change for REC1 (Movie S1, Fig. S5). The latter moves in an opposite direction with respect to REC2 and REC3, toward the HNH domain. This result agrees well with the experimental vector map of global Cas9 conformational changes, from the RNA-bound state (4ZT0.pdb) to the DNA-bound state (5F9R.pdb, Fig. S4D) (Chen *et al*., 2017). During the transition, the HNH domain forms a series of salt-bridge interactions with the REC lobe (Fig. 3). These ionic interactions mainly involve the REC1–2 regions, while HNH and REC3 remain separated from each other. This result well agrees with the novel cryo-EM structure (EMD-8236) (Huai *et al*., 2017). In this structure, HNH approaches the REC1 domain, but moves away from REC3 (Fig. S6). At ~7 µs of MD, charged residues of HNH and REC1 start engaging in ionic interactions, which increase in strength and number along the dynamics, stabilizing after ~10 µs, locking HNH at the TS (Fig. 3a). These interactions are key for the stable docking of HNH at the cleavage site. REC2 is also involved in ionic interactions, which are maintained along the simulation, indicative of the concerted conformational change of the two domains (Fig. 3b). Remarkably, interactions between HNH– REC1 are maintained in the activated state (i.e. during the last ~4 µs of MD), ensuring the positioning of HNH at the cleavage site. REC1 therefore cooperates with REC2 and REC3 in favoring HNH activation.

### Insights on the on-target specificity

**Fig. 4.**
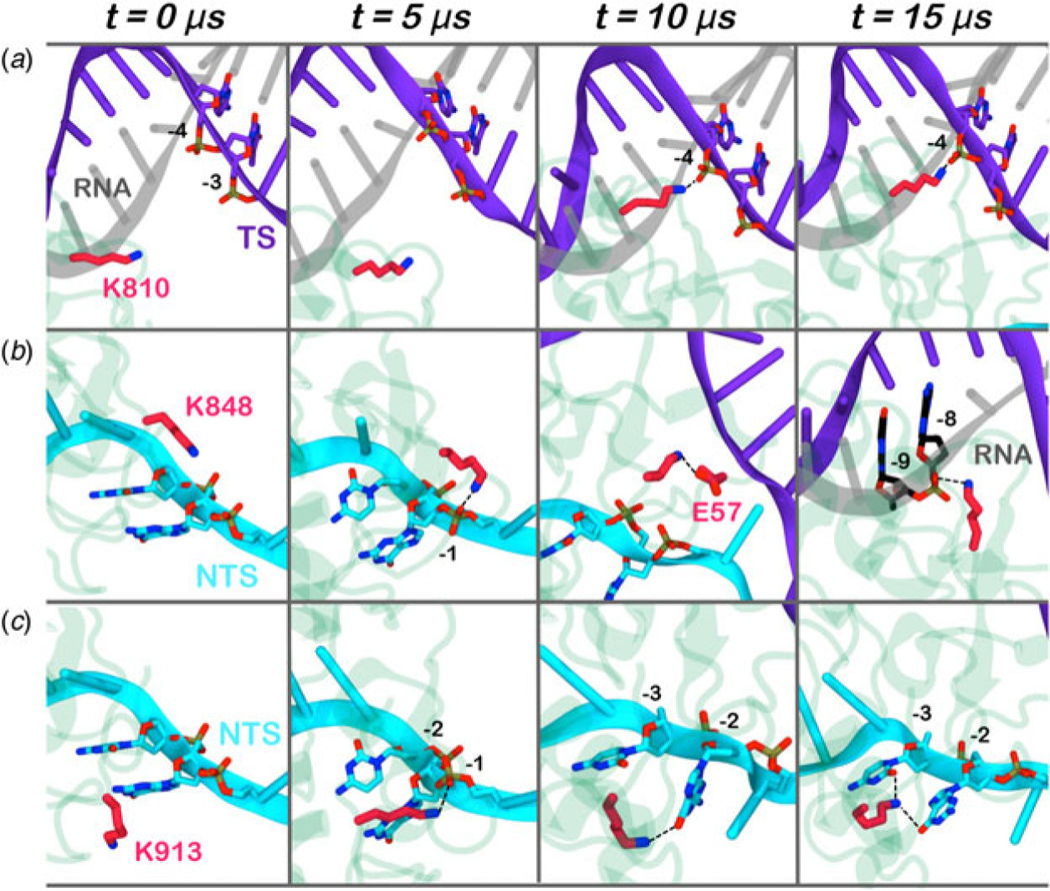
Interaction established by the K810 (a), K848 (b), and K913 (c) residues of the HNH domain at ~0, 5, 10, 15 µs of MD. The RNA (gray), target DNA (TS, magenta), and non-target DNA (NTS, cyan) strands are shown as ribbons. The HNH domain (green) is shown as cartoon. Interacting residues are shown as sticks.

Here, we monitored the dynamic interactions of key positively charged residues, which belong to the HNH domain and intervene in the specificity of CRISPR–Cas9 (Fig. 4). Among them, K810 and K848 have been shown to reduce off-target cleavage events when mutated to alanine (Slaymaker *et al*., 2016). K913 has been shown to bind the NTS in shorter time scales (i.e. ~0.8 µs), suggesting a possible role in facilitating the approach of HNH toward the scissile phosphate (Palermo *et al*., 2016). In the pre-activated state (Jiang *et al*., 2016), these residues do not interact with the nucleic acids. With the approach of HNH to the cleavage site on the TS (i.e. phosphate −3), K810 binds the phosphate −4 (Fig. 4a). Besides, K848 establishes multiple interactions with the nucleic acids and with the protein residues (Fig. 4b), finally contacting the DNA:RNA hybrid via the RNA phosphate backbone. Remarkably, in the 5Y36.pdb (all-atom model of the EMD-8236 map), K810 approaches the TS, while K848 binds the RNA backbone similarly to the configuration obtained upon ~15 µs of MD (at position −8) (Huai *et al*., 2017). K913 engages the NTS at several positions throughout the simulations (Fig. 4c). This finding confirms previous evidences from shorter MD simulations, suggesting that the interactions between the NTS and the loop formed by 906–918 residues would favor the approach of HNH toward the TS (Palermo *et al*., 2016), and also clarifies smFRET experiments showing that the docking of HNH at the TS in its active configuration requires the presence of the NTS (Dagdas *et al*., 2017). Overall, these residues act as anchors of HNH at the DNA, favoring its docking at the TS cleavage site. As such, the disruption of these interactions with K810A/K848A substitutions may destabilize HNH at the cleavage site, thereby altering the dynamics of its conformational activation and consequently its cleavage activity (Chen *et al*., 2017; Slaymaker *et al*., 2016)

### Multiple repeats along the conformational change

Continuous MD simulations of huge macromolecular systems reaching the microsecond time scale are challenging to be achieved via conventional supercomputers and the data obtained using Anton-2 are at the limit of the state-of-the-art technology. As such, the MD performed using Anton-2 in the present study, as well as in the previous studies by our and other research groups (Lindorff-Larsen *et al*., 2016; Mouchlis *et al*., 2015), enabled to capture conformational changes and biophysical processes in a single trajectory. While this approach certainly preserves the kinetic features of the system, considering the stochastic nature of the biomolecular processes, multiple simulations are required to precisely estimate the time scale of the events. However, due to the high computational cost of each single trajectory, it is challenging to produce additional and/or longer simulation runs, recovering the observed events. In order to cope with this issue and to understand the statistical relevance of the observed conformational changes, we have extracted eight equally distributed snapshots (at times 1, 3, 5, 7, 9, 11, 13, and 15 µs of the Anton-2 trajectory) to perform independent MD simulations of ~300 ns in two replicas (reaching additional ~4.8 µs of aggregate statistics). Figures S6–S8 report the time evolution along the simulated runs of the H840–P_DNA_, E60–D273, and S960–S701 distances, enabling comparison with the continuous simulation obtained with Anton-2 (Fig. 2). As a result, in the systems extracted at 1, 3, and 5 µs, in which Cas9 assumes a pre-activated configuration, the computed distances stably oscillate around the initial values. The three distances also remain stable in the activated configurations (i.e. extracted at 11, 13, and 15 µs). In the case of the systems extracted at 7 and 9 µs, at which the system undergoes the conformational transition, we observe higher fluctuations in the time evolution of the computed distances. Moreover, in the hundreds-of-nanoseconds, these latter follow the trend observed in the microsecond length dynamics, which shows the transition toward the activated state (Figs S6–S8). This highlights the tendency for the conformational transition toward activation, supporting the results obtained with single trajectory obtained with Anton-2 and previous enhanced sampling simulations (Fig. S1) (Palermo *et al*., 2017a). In order to under-stand the factors underlying this consistent behavior, we examined the structures extracted at times 1, 3, 5, 7, 9, 11, 13, and 15 µs of the Anton-2 trajectory and used as a starting point of the independent MD runs (Fig. S9). As a main difference between the pre-activated systems (i.e. extracted at times 1, 3, and 5 µs) and the system initiating the conformational transition (i.e. extracted at time 7 µs), in this latter we observe the approach of the side chains of charged residues belonging to HNH and REC1 (Fig. S9D), preluding their engagement in salt-bridge interactions, which will be fully formed at time 9 µs. At this point (9 µs, Fig. S9E), the outward transition of REC2 with respect to HNH is observed. Considering that these newly formed salt-bridge interactions are stably maintained in the activated state (i.e. for snapshots extracted at 11, 13, and 15 µs, as well as throughout the last ~6 µs of the continuous MD simulation, Fig. 3), these results suggest that the approach of the side chains of the charged residues of HNH and REC1 at ~7 µs is the first triggering event making the system prone to the larger conformational change leading to its final activation.

### Cooperative domain dynamics in the activated state

**Fig. 5.**
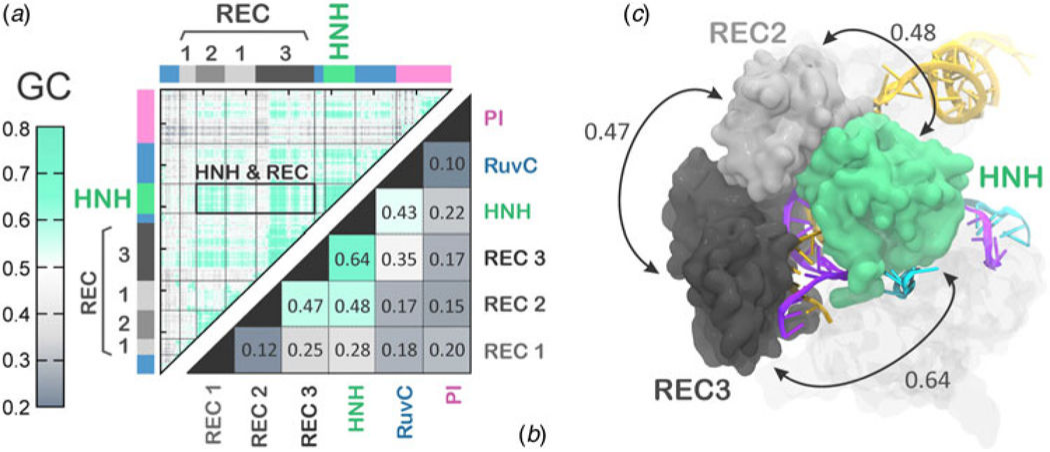
(a) Matrix of the generalized correlation (GC), calculated over the last ~4 µs of MD of CRISPR–Cas9. The strength of the computed correlations is color-coded from green (highly correlated motions) to gray (poorly correlated motions). A box is used to highlight the high correlation between the HNH domain and the REC lobe. (b) Inter-domain GC scores (GCs) plotted for each Cas9 domain (REC1–3, HNH, RuvC, and PI domain) in a two-by-two matrix, measuring the strength of the overall correlation that each domain establishes with the others (details in the Method section). (c) The highest GCs are plotted on the three-dimensional structure of CRISPR–Cas9, revealing a tight coupling between HNH and the REC2–3 domains. Cas9 is shown in a molecular surface, highlighting the HNH and REC2–3 domains with different colors. The RNA (orange) and the target DNA (TS, magenta) forming a RNA:DNA hybrid, as well as the non-target DNA (NTS, cyan) are shown as ribbons.

The activated state reached via MD simulations remains stable over the last ~6 µs (Fig. 2), allowing its conformational dynamics to be analyzed. To characterize the inter-dependent motions of the protein residues and understand the cooperative dynamics of the Cas9 domains in the activated state, we employed the Lange and Grubmüller method, which captures the overall correlations (i.e. both linear and non-linear) among protein residues (Lange and Grubmuller, 2006). The generalized correlation (GC) matrix is a sensitive method for detecting the interdependence in the motions of two spatially distant residues, and provides a measure of how much the motion of one residue is dependent on that of another residue. GC matrices have been computed for the activated configurations, over the last ~4 µs of MD. Visual inspection of the GC matrix shows that the HNH domain strongly correlates with the REC lobe in the active state (Fig. 5a). A quantitative evaluation of the inter-dependent couplings established by HNH with the REC lobe has been obtained by computing the inter-domain GC scores (GCs), which accumulate the GC coefficients of the single residues over each protein domain of interest (Fig. 5b, full details in the Methods section). The GCs are a measure of the overall inter-domain correlations, indicating the most relevant coupled motions occurring across the system during the simulations. The GCs have been useful in the characterization of the allosteric effects in CRISPR–Cas9 (Palermo *et al*., 2017b) and other protein/nucleic acid complexes (Ricci *et al*., 2016), as well as in describing the inter-dependent conformational dynamics between the protein and RNA components of the human spliceosome (Casalino *et al*., 2018). As a result, the HNH and REC3 domains display the highest overall GCs of 0.64 (Fig. 5b), which indicates a highly cooperative conformational dynamics in the activated state. Notably, both MD simulations and recent cryo-EM data (EMD-8236) (Huai *et al*.,2017) show that HNH and REC3 are not in direct contact with each other in the activated configuration (Figs 2d and 5c).Considering that the RNA:DNA hybrid locates within the groove formed by HNH and REC3 and directly interacts with both domains, the high inter-dependency of the domains motions suggests that the hybrid mediates the coupling between the two domains. This provides a plausible explanation for recent experimental observations, showing that REC3 allows for HNH nuclease activation upon recognition of the formation of the RNA: DNA hybrid (Chen *et al*., 2017). High inter-domain correlations are also detected for the HNH and REC2 (GCs of 0.48). This inter-dependent conformational dynamics corroborate the experimental evidence for tight coupling between the HNH and REC2 domains to ensure catalytic competence (Chen *et al*., 2017). Specifically, smFRET has shown reciprocal changes in the conformational states assumed by HNH and REC2 across multiple DNA substrates, indicating that the conformational dynamics of HNH and REC2 is tightly coupled to ensure catalysis. Notably, the HNH domain has high conformational plasticity, as shown by biochemical and biophysical experiments (Osuka *et al*., 2018; Sternberg *et al*., 2015) and previous shorter MD simulations (Palermo *et al*., 2016). Additionally, in the cryo-EM structure EMD-3277, the HNH and REC2 are observed at a lower resolution (8–10 Å) than the overall structure (6 Å), highlighting their mobility (Jiang *et al*., 2016). In light of this evidence, the data reported here explain how these flexible protein components are coupled to enable function. Finally, high correlations are also observed between the REC2 and REC3 (GCs of 0.47), indicating that in the activated state, the REC lobe engages in a tight interplay to enable function.

## Discussion

### Activation mechanism

In the activation process of CRISPR–Cas9, a conformational change of the catalytic HNH domain is required to trigger catalysis (Fig. 1) (Jiang *et al*., 2016). Here, MD simulations show that the docking of the HNH domain at the TS is facilitated by favorable interactions and by the conformational changes of the REC lobe. We show an opening of REC3 and an outward translation of REC2, which moves apart from its original position, resulting in the exposure of TS to the HNH active site for cleavage (Fig. 2). This result agrees well with the available experimental data (Chen and Doudna, 2017; Dagdas *et al*., 2017; Huai *et al*., 2017) and reveals that the activation of HNH is dependent on the conformational changes of the REC domains. Specifically, REC3 shows significant conformational transitions that result in the opening of the groove accommodating the RNA:DNA hybrid.This finding well agrees with recent cryo-EM data (Huai *et al*., 2017) and with the available smFRET experiments (Chen *et al*.,2017; Dagdas *et al*., 2017), showing that the activation of the HNH domain for catalysis requires a change in the conformational state of REC3. In the activated configuration, the conformational dynamics of the HNH and REC3 domains is highly coupled (Fig. 5). Considering that both HNH and REC3 directly interact with the RNA:DNA hybrid, the high inter-dependency of the domains motions suggests that the hybrid mediates the coupling between the two domains. This finding provides further clarification on recent experiments, suggesting that REC3 would allow for HNH activation upon ‘sensing’ the formation of a complete RNA:DNA hybrid (Chen *et al*., 2017). Accordingly,the simulations show that, while ‘sensing’ (i.e. interacting with) the RNA:DNA hybrid, REC3 affects the conformational dynamics of HNH and, in turn, its activation for cleavage. MD simulations also pinpoint a key role for the REC2 domain. During the conformational activation of HNH, REC2 rotates outward to enable the HNH to approach the cleavage site on the TS (Fig. 2). As well, the continuous simulation shows that during the activation process, the opening of REC2 and the approach of HNH to the TS start simultaneously (at ~7 µs, Fig. 2), indicating that the two domains cooperatively relocate to facilitate catalysis, likely in response to the ‘sensing’ of REC3 (Sung *et al*., 2018). In the activated state, HNH and REC2 show highly cooperative conformational dynamics (Fig. 5), corroborating the experimental evidence of their tight coupling to ensure catalytic competence (Chen *et al*., 2017). Taken together, these findings suggest that REC2 functions as a ‘regulator’ for HNH function (Chen and Doudna, 2017; Chen *et al*., 2017). MD simulations further reveal an unexpected role for REC1, which approaches HNH by establishing a series of ionic interactions, thereby enabling HNH to dock at the cleavage site on the TS. These observations suggest that REC1 acts as a ‘lock’ for HNH (Fig. 3). Overall, the tight interplay observed among the REC1–3 domains and the HNH domain reveals how the ‘sensor’ (REC3), the ‘regulator’ (REC2) of HNH activation (Chen and Doudna, 2017; Chen *et al*., 2017), together with the ‘lock’ (REC1), contribute to the formation of an activated CRISPR–Cas9 complex (Movie S2).

### Conclusions

Here, we disclose the mechanism leading to the formation of a catalytically active CRISPR–Cas9 nuclease following substrate DNA binding. Unbiased all-atoms MD simulations reveal the approach of the HNH domain to the cleavage site on the TS in ~16 μs of continuous simulation. We characterize on an atomic scale the molecular determinants leading to the HNH conformational transition and the key role of the REC lobe in enabling the docking of HNH at the cleavage site. The remarkable conformational changes and the collective conformational dynamics of the REC1–3 domains enable ‘sensing’ of the nucleic acid binding, ‘regulation’ of the HNH conformational transition and ‘locking’ of the HNH at the cleavage site, eventually leading to the formation of a catalytically active CRISPR–Cas9 (Movie S2). Good agreement with biochemical experiments and structural data highlights the consistency of the activated conformation. As such, these simulations add detailed mechanistic information on the CRISPR–Cas9 activation process, clarifying the mechanistic role of the REC lobe components. Considering the key role of the REC lobe in the specificity of Cas9 (Casini *et al*., 2018; Chen *et al*., 2017; Kleinstiver *et al*., 2016), this study provides the foundation for understanding how the REC lobe domains control the cleavage of off-target sequences. Toward this aim, extensive characterization is currently ongoing in our laboratories. Overall, the knowledge on the HNH activation process and the role of the recognition lobe components, deciphered here, establishes a framework for future studies and novel structure-based engineering efforts for improved genome editing

## Material and method section

### Structural model

MD simulations were based on the X-ray structure of the S. pyogenes Cas9 in complex with RNA and DNA (5F9R.pdb), solved at 3.40 Å resolution (Jiang *et al*., 2016). The model system was embedded in explicit waters, while Na+ ions were added to neutralize the total charge, leading to an orthorhombic periodic simulation cell of ~180 · 120 · 140 Å^3^, for a total of ~ 300 000 atoms.

### MD simulations

A simulation protocol tailored for RNA/DNA endonucleases was adopted (Palermo *et al*., 2015; Sponer *et al*., 2018), embracing the use of the Amber ff12SB force field, which includes the ff99bsc0 corrections for DNA (Perez *et al*., 2007) and the ff99bsc0 + *χOL3* corrections for RNA (Banas *et al*., 2010; Zgarbova *et al*., 2011). The TIP3P model was employed for explicit water molecules (Jorgensen *et al*., 1983). The Åqvist (Aqvist, 1990) force field was employed for Mg ions, as in previous studies on similar Mg-aided RNA/DNA nucleases (Casalino *et al*., 2016; 2017; Palermo *et al*., 2013). An integration time step of 2 fs was used. All bond lengths involving hydrogen atoms were constrained using the SHAKE algorithm (Ryckaert *et al*., 1977). Temperature control (300 K) was performed via Langevin dynamics (Turq *et al*., 1977), with a collision frequency *γ* = 1. Pressure control was accomplished by coupling the system to a Berendsen barostat (Berendsen *et al*., 1984), at a reference pressure of 1 atm and with a relaxation time of 2 ps. The system was subjected to energy minimization to relax water molecules and counter ions, keeping the protein, the RNA, DNA, and Mg ions fixed with harmonic position restraints of 300 kcal/mol · Å^2^. Then, the system was heated up from 0 to 100 K in the canon-canonical ensemble (NVT), by running two simulations of 5 ps each, imposing position restraints of 100 kcal/mol · Å^2^ on the abovementioned elements of the system. The temperature was further increased up to 200 K in ~100 ps of MD in the isothermal–isobaric ensemble (NPT), reducing the restraint to 25 kcal/mol · Å^2^. Subsequently, all restraints were released and the temperature of the system was raised up to 300 K in a single NPT simulation of 500 ps. After ~1.1 ns of equilibration, ~10 ns of NPT runs were carried out allowing the density of the system to stabilize around 1.01 g/cm^3^. Finally, the production runs were carried out in the NVT ensemble. Simulations were performed using the GPU version of AMBER 16 (Case *et al*., 2016; Salomon-Ferrer *et al*.,2013) and the SPFP precision model (Le Grand *et al*., 2013). MD simulations were performed to equilibrate the system for ~120 ns prior to long time scale continuous MD. Indeed, the wellequilibrated system was used as a starting point for simulations on Anton-2 (described below). As well, eight equally distributed snapshots were extracted at times 1, 3, 5, 7, 9, 11, 13, and 15 μs of the Anton-2 trajectory and subjected to independent MD simulations in two replicas (i.e. ~300 ns for each snapshot in two replicas), reaching a total of additional 4.8 μs.

Long time scale MD simulations of the CRISPR–Cas9 complex were performed using Anton-2 (Shaw *et al*., 2014), a specialpurpose supercomputer for micro-to-millisecond length MD, starting from a well-equilibrated configuration, obtained after ~120 ns of conventional MD (see above). Simulations on Anton-2 were performed using the same force-field parameters used for conventional MD simulations. A reversible multiple time step algorithm (Tuckerman *et al*., 1992) was employed to integrate the equations of motion with a time step of 2 fs for short-range non-bonded and bonded forces and 6 fs for the long range non-bonded forces, for a total of ~16 μs of simulations. Simulations were performed at constant temperature (300 K) and pressure (1 atm) using the multigrator integrator as implemented in Anton-2 (Lippert *et al*., 2013). The k-Gaussian split Ewald method (Shan *et al*., 2005) was used for long-range electrostatic interactions. Hydrogen atoms were added assuming standard bond lengths and were constrained to their equilibrium position with the SHAKE algorithm (Ryckaert *et al*., 1977). Overall, molecular simulations have been carried out for an over-all sampling time of >20 µs.

### Principal component analysis

In PCA, the covariance matrix of the protein *Cα* atoms is calculated and diagonalized to obtain a new set of generalized coordinates (eigenvectors) to describe the system motions. Each eigenvector, called Principal Component (PC), is associated to an eigenvalue corresponding to the mean square fluctuation contained in the system’s trajectory projected along that eigenvector. The first PC1 corresponds to the system’s largest amplitude motion, and the dynamics of the system along PC1 is usually referred to as ‘essential dynamics’ (Amadei *et al*., 1993). In this work, each conformation sampled during MD was projected into the collective coordinate space defined by the first two eigen-vectors (PC1 and PC2), characterizing the essential conformational sub-space sampled by Cas9. Full details are in the SI.

### Correlation analysis

Cross-correlations of residues in the Cas9 protein were computed based on mutual information between all *Cα* atoms using the generalized correlation analysis approach developed by Lange and Grubmüller (Lange and Grubmuller, 2006), which is explained in detail in the SI. The g_correlation module in the Gromacs 3.3 (Lindahl *et al*., 2001) package has been employed. Based on the computed correlations, the Generalized Correlation score (GCs) has been employed to define quantitatively the inter-dependent couplings established by HNH with the REC lobe. The GCs has been originally introduced by Ricci *et al*. in the study of the conformational dynamics of the PPAR*γ*–RXR*α* nuclear receptor complex (Ricci *et al*., 2016), and used to describe the allosteric effects in CRISPR–Cas9 (Palermo *et al*., 2017b) and the inter-dependent dynamics of the human spliceosome (Casalino *et al*., 2018). The GCs are computed by processing the GC matrix as follows. For each amino acid residue a GCs can be defined:

(1) $${\text{GCs}}_{i}=\sum_{j\neq i}^{N}{\text{GC}}_{ij},$$

representing a measure of both the number and the intensity of the GC coefficients displayed by each residue. To filter non-trivial correlations and eliminate the noise due to uncorrelated motions, per-residue GCs were computed considering only highly positive correlations (GC ≥ 0.60). GCs were used to detail the overall inter-domain correlations as follows: GCs were calculated for each residue i belonging to a specific protein domain (e.g. HNH, Rec1–3), with the residues j belonging to another protein domain of interest. Then, GCs were accumulated over all residues j of each specific Cas9 domain and normalized by the number of coupling residues i and j, which display GC ≥ 0.60. This resulted in a set of per-domain GCs, ranging from 0 (not-correlated) to 1 (correlated), measuring the strength of the overall correlation that each domain establishes with the others. The overall GCs are a measure of the most important correlated motions among protein domains taking place in the simulations, which help in identifying how specific protein regions mechanistically intervene in the overall correlation network (Palermo *et al*., 2017b; Ricci *et al*., 2016).

## Supplementary Material

Movie S1

Movie S2

SI

## References

[R1] Abrams C and Bussi G (2014) Enhanced sampling in molecular dynamics using metadynamics, replica-exchange, and temperature-acceleration. Entropy 16, 163–199.

[R2] Amadei A, Linssen ABM and Berendsen HJC (1993) Essential dynamics of proteins. Proteins-Structure Function and Genetics 17, 412–425. 10.1002/prot.340170408 8108382

[R3] Anders C, Niewoehner O, Duerst A and Jinek M (2014) Structural basis of PAM-dependent target DNA recognition by the Cas9 endonuclease. Nature 513, 569–573. 25079318 10.1038/nature13579 PMC4176945

[R4] Aqvist J (1990) Ion-water interaction potentials derived from free energy per-turbation simulations. Journal of Physical Chemistry 94, 8021–8024.

[R5] Banas P, Hollas D, Zgarbova M, Jurecka P, Orozco M, Cheatham TE, Sponer J and Otyepka M (2010) Performance of molecular mechanics force fields for RNA simulations: stability of UUCG and GNRA hairpins. Journal of Chemical Theory and Computation 6, 3836–3849. 10.1021/ct100481h PMC8916691 35283696

[R6] Berendsen HJC, Postma JPM, Van Gunsteren WF, Dinola A and Haak JR (1984) Molecular dynamics with coupling to an external bath. The Journal of Chemical Physics 81, 3684–3690.

[R7] Casalino L, Palermo G, Abdurakhmonova N, Rothlisberger U and Magistrato A (2017) Development of site-specific Mg2+-RNA force field parameters: a dream or reality? Guidelines from combined molecular dynamics and quantum mechanics simulations. Journal of Chemical Theory and Computation 13, 340–352. 28001405 10.1021/acs.jctc.6b00905

[R8] Casalino L, Palermo G, Rothlisberger U and Magistrato A (2016) Who activates the nucleophile in ribozyme catalysis? An answer from the splicing mechanism of group II introns. Journal of the American Chemical Society 138, 10374–10377. 27309711 10.1021/jacs.6b01363

[R9] Casalino L, Palermo G, Spinello A, Rothlisberger U and Magistrato A (2018) All-atom simulations disentangle the functional dynamics underlying gene maturation in the intron lariat spliceosome. Proceedings of the National Academy of Sciences of the USA 115, 6584–6589. 29891649 10.1073/pnas.1802963115 PMC6042132

[R10] Case DA, Betz RM, Botello-Smith W, Cerutti DS, Cheatham TE III, Darden TA, Duke RE, Giese TJ, Gohlke H, Goetz AW, Homeyer N, Izadi S, Janowski P, Kaus J, Kovalenko A, Lee TS, Legrand S, Li P, Lin C, Luchko T, Luo R, Madej B, Mermelstein D, Merz KM, Monard G, Nguyen H, Nguyen HT, Omelyan I, Onufriev A, Roe DR, Roitberg A, Sagui C, Simmerling CL, Swails J, Walker RC, Wang J, Wolf RM, Wu X, Xiao L, York DM and Kollman PA (2016). AMBER 2016. San Francisco: University of California

[R11] Casini A, Olivieri M, Petris G, Montagna C, Reginato G, Maule G, Lorenzin F, Prandi D, Romanel A, Demichelis F, Inga A and Cereseto A (2018) A highly specific spCas9 variant is identified by in vivo screening in yeast. Nature Biotechnology 36, 265–271. 10.1038/nbt.4066 PMC6066108 29431739

[R12] Chen JS, Dagdas YS, Kleinstiver BP, Welch MM, Harrington LB, Sternberg SH, Joung JK, Yildiz A and Doudna JA (2017) Enhanced proof-reading governs CRISPR-Cas9 targeting accuracy. Nature 550, 407–410. 28931002 10.1038/nature24268 PMC5918688

[R13] Chen JS and Doudna JA (2017) The chemistry of Cas9 and its CRISPR colleagues. Nature Reviews Chemistry 1, 78.

[R14] Dagdas YS, Chen JS, Sternberg SH, Doudna JA and Yildiz A (2017) A conformational checkpoint between DNA binding and cleavage By CRISPR-Cas9. Science Advances 3, eaao0027. 28808686 10.1126/sciadv.aao0027 PMC5547770

[R15] Doudna JA and Charpentier E (2014) Genome editing. The new frontier of genome engineering with CRISPR-Cas9. Science 346, 1258096–1258099. 25430774 10.1126/science.1258096

[R16] Huai C, Li G, Yao RJ, Zhang Y, Cao M, Kong LL, Jia CQ, Yuan H, Chen HY, Lu DR and Huang Q (2017) Structural insights into DNA cleavage activation of CRISPR-Cas9 system. Nature Communications 8, 1375. 10.1038/s41467-017-01496-2 PMC5680257 29123204

[R17] Jiang F and Doudna JA (2017) CRISPR-Cas9 structures and mechanisms. Annual Review of Biophysics 46, 505–529. 10.1146/annurev-biophys-062215-010822 28375731

[R18] Jiang F, Zhou K, Ma L, Gressel S and Doudna JA (2015) STRUCTURAL BIOLOGY. A Cas9-guide RNA complex preorganized for target DNA recognition. Science 348, 1477–1481. 26113724 10.1126/science.aab1452

[R19] Jiang FG, Taylor DW, Chen JS, Kornfeld JE, Zhou KH, Thompson AJ, Nogales E and Doudna JA (2016) Structures of a CRISPR-Cas9 R-loop complex primed for DNA cleavage. Science 351, 867–871. 26841432 10.1126/science.aad8282 PMC5111852

[R20] Jinek M, Chylinski K, Fonfara I, Hauer M, Doudna JA and Charpentier E (2012) A programmable dual-RNA-guided DNA endonuclease in adaptive bacterial immunity. Science 337, 816–821. 22745249 10.1126/science.1225829 PMC6286148

[R21] Jinek M, Jiang F, Taylor DW, Sternberg SH, Kaya E, Ma E, Anders C, Hauer M, Zhou K, Lin S, Kaplan M, Iavarone AT, Charpentier E, Nogales E and Doudna JA (2014) Structures of Cas9 endonucleases reveal RNA-mediated conformational activation. Science 343, 1247997–1247111. 24505130 10.1126/science.1247997 PMC4184034

[R22] Jorgensen WL, Chandrasekhar J, Madura JD, Impey RW and Klein ML (1983) Comparison of simple potential functions for simulating liquid water. Journal of Chemical Physics 79, 926–935.

[R23] Kleinstiver BP, Pattanayak V, Prew MS, Tsai SQ, Nguyen NT, Zheng ZL and Joung JK (2016) High-fidelity CRISPR-Cas9 nucleases with no detect-able genome-wide off-target effects. Nature 529, 490–595. 26735016 10.1038/nature16526 PMC4851738

[R24] Lange OF and Grubmuller H (2006) Generalized correlation for biomolecular dynamics. Proteins-Structure Function and Bioinformatics 62, 1053–1061. 10.1002/prot.20784 16355416

[R25] Le Grand S, Goetz AW and Walker RC (2013) SPFP: speed without compromise – a mixed precision model for GPU accelerated molecular dynamics simulations. Computer Physics Communications 148, 374–380.

[R26] Lindahl E, Hess B and Van Der Spoel D (2001) GROMACS 3.0: a package for molecular simulation and trajectory analysis. Journal of Molecular Modeling 7, 306–317.

[R27] Lindorff-Larsen K, Maragakis P, Piana S and Shaw DE (2016) Picosecond to millisecond structural dynamics in human ubiquitin. The Journal of Physical Chemistry B 120, 8313–8320. 27082121 10.1021/acs.jpcb.6b02024

[R28] Lippert RA, Predescu C, Ierardi DJ, Mackenzie KM, Eastwood MP, Dror RO and Shaw DE (2013) Accurate and efficient integration for molecular dynamics simulations at constant temperature and pressure. The Journal of Chemical Physics 139. 10.1063/1.4825247 24182003

[R29] Miao Y, Feher VA and Mccammon JA (2015) Gaussian accelerated molecular dynamics: unconstrained enhanced sampling and free energy calculation. Journal of Chemical Theory and Computation 11, 3584–3595. 26300708 10.1021/acs.jctc.5b00436 PMC4535365

[R30] Miao Y and Mccammon JA (2016a) Graded activation and free energy landscapes of a muscarinic G protein-coupled receptor. Proceedings of the National Academy of Sciences of the USA 113, 12162–12167. 27791003 10.1073/pnas.1614538113 PMC5087018

[R31] Miao Y and Mccammon JA (2016b) Unconstrained enhanced sampling for free energy calculations of biomolecules: a review. Molecular Simulations 42, 1046–1055. 10.1080/08927022.2015.1121541 PMC4955644 27453631

[R32] Miao Y and Mccammon JA (2018) Mechanism of the G-protein mimetic nano-body binding to a muscarinic G-protein-coupled receptor. Proceedings of the National Academy of Sciences of the USA 115, 3036–3041. 29507218 10.1073/pnas.1800756115 PMC5866610

[R33] Mouchlis VD, Bucher D, Mccammon JA and Dennis EA (2015) Membranes serve as allosteric activators of phospholipase A(2), enabling it to extract, bind, and hydrolyze phospholipid substrates. Proceedings of the National Academy of Sciences of the USA 112, E516–E525. 25624474 10.1073/pnas.1424651112 PMC4330758

[R34] Nishimasu H and Nureki O (2017) Structures and mechanisms of CRISPR RNA-guided effector nucleases. Current Opinion in Structural Biology 43, 68–78. 27912110 10.1016/j.sbi.2016.11.013

[R35] Nishimasu H, Ran FA, Hsu PD, Konermann S, Shehata SI, Dohmae N, Ishitani R, Zhang F and Nureki O (2014) Crystal structure of Cas9 in complex with guide RNA and target DNA. Cell 156, 935–949. 24529477 10.1016/j.cell.2014.02.001 PMC4139937

[R36] Nogales E (2016) The development of cryo-EM into a mainstream structural biology technique. Nature Methods 13, 24–27. 27110629 10.1038/nmeth.3694 PMC4913480

[R37] Osuka S, Isomura K, Kajimoto S, Komori T, Nishimasu H, Shima T, Nureki O and Uemura S (2018) Real-time observation of flexible domain movements in Cas9. The EMBO Journal e96941. 29650679 10.15252/embj.201796941 PMC5978321

[R38] Palermo G, Cavalli A, Klein ML, Alfonso-Prieto M, Dal Peraro M and De Vivo M (2015) Catalytic metal ions and enzymatic processing of DNA and RNA. Accounts of Chemical Research 48, 220–228. 25590654 10.1021/ar500314j

[R39] Palermo G, Miao Y, Walker RC, Jinek M and Mccammon JA (2016) Striking plasticity of CRISPR-Cas9 and key role of non-target DNA, as revealed by molecular simulations. ACS Central Science 2, 756–763. 27800559 10.1021/acscentsci.6b00218 PMC5084073

[R40] Palermo G, Miao Y, Walker RC, Jinek M and Mccammon JA (2017a) CRISPR-Cas9 conformational activation as elucidated from enhanced molecular simulations. Proceedings of the National Academy of Sciences of the USA 114, 7260–7265. 28652374 10.1073/pnas.1707645114 PMC5514767

[R41] Palermo G, Ricci CG, Fernando A, Rajshekhar B, Jinek M, Rivalta I, Batista VS and Mccammon JA (2017b) PAM-induced allostery activates CRISPR-Cas9. Journal of the American Chemical Society 139, 16028–16031. 28764328 10.1021/jacs.7b05313 PMC5905990

[R42] Palermo G, Stenta M, Cavalli A, Dal Peraro M and De Vivo M (2013) Molecular simulations highlight the role of metals in catalysis and inhibition of type II opoisomerase. Journal of Chemical Theory and Computation 9, 857–862. 26588728 10.1021/ct300691u

[R43] Paul F, Wehmeyer C, Abualrous ET, Wu H, Crabtree MD, Schoneberg J, Clarke J, Freund C, Weikl TR and Noé F (2017) Protein-peptide association kinetics beyond the seconds timescale from atomistic simulations. Nature Communications 8, 1095. 10.1038/s41467-017-01163-6 PMC5653669 29062047

[R44] Perez A, Marchan I, Svozil D, Sponer J, Cheatham TE III, Laughton CA and Orozco M (2007) Refinement of the AMBER force field for nucleic acids: improving the description of alpha/gamma conformers. Biophysical Journal 92, 3817–3829. 17351000 10.1529/biophysj.106.097782 PMC1868997

[R45] Raper AT, Stephenson AA and Suo Z (2018) Functional insights revealed by the kinetic mechanism of CRISPR/Cas9. Journal of the American Chemical Society 140, 2971–2984. 29442507 10.1021/jacs.7b13047

[R46] Ricci CG, Silveira RL, Rivalta I, Batista VS and Skaf MS (2016) Allosteric pathways in the PPAR gamma-RXR alpha nuclear receptor complex. Scientific Reports 6, 19940. 26823026 10.1038/srep19940 PMC4731802

[R47] Ryckaert JP, Ciccotti G and Berendsen HJC (1977) Numerical-integration of Cartesian equations of motion of a system with constraints – molecular-dynamics of N-alkanes. Journal of Computational Physics 23, 327–341.

[R48] Salomon-Ferrer R, Gotz AW, Poole D, Le Grand S and Walker RC (2013) Routine microsecond molecular dynamics simulations with AMBER on GPUs. 2. Explicit solvent particle mesh Ewald. Journal of Chemical Theory and Computation 9, 3878–3888. 26592383 10.1021/ct400314y

[R49] Shan YB, Klepeis JL, Eastwood MP, Dror RO and Shaw DE (2005) Gaussian split Ewald: a fast Ewald mesh method for molecular simulation. Journal of Chemical Physics 122, 54101. 15740304 10.1063/1.1839571

[R50] Shaw DE, Grossman JP, Bank JA, Batson B, Butts JA, Chao JC and Deneroff MM (2014) Anton 2: raising the bar for performance and pro-grammability in a special-purpose molecular dynamics supercomputer 41–53. 10.1109/SC.2014.9.

[R51] Shibata M, Nishimasu H, Kodera N, Hirano S, Ando T, Uchihashi T and Nureki O (2017) Real-space and real-time dynamics of CRISPR-Cas9 visualized by high-speed atomic force microscopy. Nature Communications 8, 1430. 10.1038/s41467-017-01466-8 PMC5681550 29127285

[R52] Singh D, Sternberg SH, Fei J, Doudna JA and Ha T (2016) Real-time observation of DNA recognition and rejection by the RNA-guided endonuclease Cas9. Nature Communications 7, 12778. 10.1038/ncomms12778 PMC5027287 27624851

[R53] Slaymaker IM, Gao L, Zetsche B, Scott DA, Yan WX and Zhang F (2016) Rationally engineered Cas9 nucleases with improved specificity. Science 351, 84–88. 26628643 10.1126/science.aad5227 PMC4714946

[R54] Sponer J, Bussi G, Krepl M, Banas P, Bottaro S, Cuhna RA, Gil-Ley A, Pinamonti G, Poblete S, Jurecka P, Walter NG and Otyepka M (2018) RNA structural dynamics as captured by molecular simulations: a comprehensive overview. Chemical Reviews 118, 4177–4338. 29297679 10.1021/acs.chemrev.7b00427 PMC5920944

[R55] Stelzl LS and Hummer G (2017) Kinetics from replica exchange molecular dynamics simulations. Journal of Chemical Theory and Computations 13, 3927–3935. 10.1021/acs.jctc.7b00372 28657736

[R56] Sternberg SH, Lafrance B, Kaplan M and Doudna JA (2015) Conformational control of DNA target cleavage by CRISPR-Cas9. Nature 527, 110–113. 26524520 10.1038/nature15544 PMC4859810

[R57] Sung K, Park J, Kim J, Lee LK and Kim SK (2018) Target specificity of Cas9 nuclease via DNA rearrangement regulated by the REC2 domain. Journal of the American Chemical Society 140, 7778–7781. 29874063 10.1021/jacs.8b03102

[R58] Tuckerman M, Berne BJ and Martyna GJ (1992) Reversible multiple time scale molecular-dynamics. Journal of Chemical Physics 97, 1990–2001.

[R59] Turq P, Lantelme F and Friedman HL (1977) Brownian dynamics – its application to ionic-solutions. Journal of Chemical Physics 66, 3039–3044.

[R60] Zgarbova M, Otyepka M, Sponer J, Mladek A, Banas P, Cheatham TE, Jurecka P (2011) Refinement of the Cornell Nucleic acids force field based on reference quantum chemical calculations of glycosidic torsion profiles. Journal of Chemical Theory and Computation 7, 2886–2902. 21921995 10.1021/ct200162x PMC3171997

[R61] Zuo Z and Liu J (2017) Structure and dynamics of Cas9 HNH domain catalytic state. Scientific Reports 7, 17271. 29222528 10.1038/s41598-017-17578-6 PMC5722908

[R62] Palermo G, Chen JS, Ricci CG, Rivalta I, Jinek M, Batista VS, Doudna JA, McCammon JA (2018). Key role of the REC lobe during CRISPR–Cas9 activation by ‘sensing’, ‘regulating’, and ‘locking’ the catalytic HNH domain. Quarterly Reviews of Biophysics 51, e9, 1–11. https://doi.org/10.1017/S0033583518000070 10.1017/S0033583518000070 PMC6292676 30555184

