# Supplementary material for: Key role of the REC lobe during CRISPR–Cas9 activation by ‘sensing’, ‘regulating’, and ‘locking’ the catalytic HNH domain
Source: Q Rev Biophys. Author manuscript; Available in PMC 2018 Dec 13. (PMC6292676; doi:10.1017/S0033583518000070)
Supplement: SI [file NIHMS992633-supplement-SI.docx]

**Formation of an activated Cas9 by “sensing”, “regulating” and “locking” the catalytic HNH domain**

Giulia Palermo, Janice S. Chen, Clarisse G. Ricci, Ivan Rivalta, Martin Jinek, Victor S. Batista, Jennifer A. Doudna and J. Andrew McCammon

**SUPPLEMENTARY INFORMATION**

**1.** SUPPLEMENTARY MATERIALS AND METHODS...………………..pp. S2-S6

**2.** SUPPLEMENTARY FIGURES (9) ……………………………………pp. S7-S16

**3.** SUPPLEMENTARY MOVIES (2) ………………..………………..………p. S17

**4.** SUPPLEMENTARY REFERENCES (35)…………………………….pp. S18-S20

Corresponding authors:

Dr. Giulia Palermo ([gpalermo@ucsd.edu](mailto:gpalermo@ucsd.edu))

**1. Supplementary Materials and Methods**

**1.1. Structural model**

MD simulations have been based on the X-ray structure of the *Streptococcus pyogenes* Cas9 in complex with RNA and DNA (5F9R.pdb), solved at 3.40 Å resolution ([Jiang et al., 2016](#_ENREF_12)). The model system has been embedded in explicit waters, while Na+ ions were added to neutralize the total charge, leading to an orthorhombic periodic simulation cell of ~180 · 120 · 140 Å^3^, for a total of ~300,000 atoms. The pre-activated state of CRISPR-Cas9 has been crystallized in the absence of the catalytic Mg metal ions ([Jiang et al., 2016](#_ENREF_12)). However, given the importance of Mg ions during the catalytic process, catalytic Mg ions have been included in the RuvC and HNH active sites, as done in Jinek et al. ([Jinek et al., 2014](#_ENREF_13)).

**1.2. Molecular Dynamics (MD) simulations**

MD simulations have been performed to equilibrate the system prior to long timescale continuous MD. A simulation protocol tailored for RNA/DNA endonucleases has been adopted ([Palermo et al., 2015](#_ENREF_19); [Sponer et al., 2018](#_ENREF_30)), embracing the use of the Amber ff12SB force field, which includes the ff99bsc0 corrections for DNA ([Perez et al., 2007](#_ENREF_23)) and the ff99bsc0+*χOL3* corrections for RNA ([Banas et al., 2010](#_ENREF_3); [Zgarbova et al., 2011](#_ENREF_35)). The TIP3P model has been employed for explicit water molecules ([Jorgensen et al., 1983](#_ENREF_14)). The Åqvist ([Aqvist, 1990](#_ENREF_2)) force field has been employed for Mg ions, as in previous studies on similar Mg-aided RNA/DNA nucleases ([Casalino et al., 2017](#_ENREF_5); [Casalino et al., 2016](#_ENREF_6); [Palermo et al., 2013](#_ENREF_22)). An integration time step of 2 fs has been employed. All bond lengths involving hydrogen atoms were constrained using the SHAKE algorithm ([Ryckaert et al., 1977](#_ENREF_26)). Temperature control (300 K) has been performed via Langevin dynamics ([Turq et al., 1977](#_ENREF_33)), with a collision frequency γ = 1. Pressure control was accomplished by coupling the system to a Berendsen barostat ([Berendsen et al., 1984](#_ENREF_4)), at a reference pressure of 1 atm and with a relaxation time of 2 ps. The system has been subjected to energy minimization to relax water molecules and counter ions, keeping the protein, the RNA, DNA and Mg ions fixed with harmonic position restraints of 300 kcal/mol · Å^2^. Then, the system has been heated up from 0 to 100 K in the canonical ensemble (NVT), by running two simulations of 5 ps each, imposing position restraints of 100 kcal/mol · Å^2^ on the above-mentioned elements of the system. The temperature was further increased up to 200 K in ~100 ps of MD in the isothermal-isobaric ensemble (NPT), reducing the restraint to 25 kcal/mol · Å^2^. Subsequently, all restraints were released and the temperature of the system was raised up to 300 K in a single NPT simulation of 500 ps. After ~ 1.1 ns of equilibration, ~ 10 ns of NPT runs were carried out allowing the density of the system to stabilize around 1.01 g/cm^-3^. Finally, the production run was carried out in the NVT ensemble, collecting ~120 ns. Simulations have been performed using the GPU version of AMBER 16 ([Case et al., 2016](#_ENREF_7); [Salomon-Ferrer et al., 2013](#_ENREF_27)) and the SPFP precision model ([Le Grand et al.](#_ENREF_16)). The well-equilibrated system has been used as a starting point for simulations on Anton-2.

Long timescale MD simulations of the CRISPR-Cas9 complex have been performed using Anton-2 ([Shaw et al., 2014](#_ENREF_29)), a special-purpose supercomputer for micro-to-millisecond length MD simulations, starting from a well-equilibrated configuration, obtained after ~120 ns of conventional MD (see above). Simulations on Anton-2 have been performed using the same force field parameters used for conventional MD simulations. A reversible multiple time step algorithm ([Tuckerman et al., 1992](#_ENREF_32)) was employed to integrate the equations of motion with a time step of 2 fs for short-range nonbonded and bonded forces and 6 fs for the long-range nonbonded forces, for a total of ∼16 μs of simulations. Simulations were performed at constant temperature (300 K) and pressure (1 atm) using the multigrator integrator as implemented in Anton-2 ([Lippert et al., 2013](#_ENREF_18)). The k-Gaussian split Ewald method ([Shan et al., 2005](#_ENREF_28)) was used for long-range electrostatic interactions. Hydrogen atoms were added assuming standard bond lengths and were constrained to their equilibrium position with the SHAKE algorithm ([Ryckaert et al., 1977](#_ENREF_26)).

**1.3. Principal Component Analysis (PCA)**

In PCA, the covariance matrix of the protein Cα atoms is calculated and diagonalized to obtain a new set of generalized coordinates (eigenvectors) to describe the system motions. Each eigenvector – also called Principal Component (PC) – is associated to an eigenvalue corresponding to the mean square fluctuation contained in the system’s trajectory projected along that eigenvector. By sorting the eigenvectors according to their eigenvalues, the first Principal Component (PC1) corresponds to the system’s largest amplitude motion, and the dynamics of the system along PC1 is usually referred as *“essential dynamics”* ([Amadei et al., 1993](#_ENREF_1)). In this work, each conformation sampled during the MD trajectories is projected into the collective coordinate space defined by the first two eigenvectors (PC1 and PC2), such allowing the characterization of the essential conformational sub-space sampled by Cas9 during MD. PCA has been performed using the GROMACS 4.4.5 suite of analysis codes ([Van der Spoel et al., 2005](#_ENREF_34)). In detail, the g_covar program has been employed for the construction and diagonalization of the covariance matrix. Subsequently, the program g_anaeig has been used to analyze and visualize the eigenvectors. Fig. S5 has been produced using the Normal Mode Wizard (NMWiz) plugin of the Visual Molecular Dynamics (VMD) molecular visualization program ([Humphrey et al., 1996](#_ENREF_11)).

**1.4. Generalized Correlations (GC) analysis**

Generalized Correlations (GC) analysis was used to capture dynamic correlations in the simulated systems. With respect to the more traditional Pearson coefficients analysis, GC analysis has the advantage of capturing correlations independently of the relative orientation of the atomic fluctuations, while also being able to capture non-linear correlations ([Lange & Grubmuller, 2006](#_ENREF_15)). Two variables (x*_i_*,x*_j_*) can be considered correlated when their joint probability distribution, $p\left( x_{i}, x_{j} \right),$ is smaller than the product of their marginal distributions,$p\left( x_{i} \right)\cdot p\left( x_{j} \right)$. The mutual information ($MI$) is a measure of the degree of correlation between $x_{i}$and $x_{j}$defined as function of $p\left( x_{i}, x_{j} \right)$ and $p\left( x_{i} \right)\cdot p\left( x_{j} \right)$ according to:

$MI \left[ x_{i}, x_{j} \right]= \iint p\left( x_{i}, x_{j} \right)\ln\frac{p\left( x_{i}, x_{j} \right)}{p\left( x_{i} \right)\cdot p\left( x_{j} \right)}dx_{i}dx_{j}$ [1]

Notably, *MI* is closely related to the definition of the Shannon entropy, H[x], i.e., the expectation value of a random variable $x$, having a probability distribution $p\left( x_{i} \right):$

$H\left[ x \right]= \int p\left( x \right)\ln p\left( x \right)dx$ [2]

and it can be thus computed as:

$MI \left[ x_{i}, x_{j} \right]= H \left[ x_{i} \right]+ H \left[ x_{j} \right]- H \left[ x_{i}{, x}_{j} \right]$ [3]

where $H \left[ x_{i} \right]$ and $H \left[ x_{j} \right]$ are the marginal Shannon entropies, and $H \left[ x_{i}{, x}_{j} \right]$is the joint entropy. The g_correlation tool ([Lange & Grubmuller, 2006](#_ENREF_15)), implemented in Gromacs 3.3 ([Lindahl et al., 2001](#_ENREF_17)), was used to calculate the marginal entropies$H \left[ x_{i} \right]$ and $H \left[ x_{j} \right]$ and the joint entropy$H \left[ x_{i}{, x}_{j} \right]$ by means of the *k*-nearest neighbor distances algorithm ([Floyd, 1962](#_ENREF_9)), applied to the atomic position fluctuations from MD simulations. Since $MI$ varies from 0 to + ∞, normalized GC coefficients, ranging from 0 (independent variables) to 1 (fully correlated variables), are defined as:

${GC}_{ij} \left[ x_{i}, x_{j} \right]=\left\{ 1- e^{-2MI\left[ x_{i}, x_{j} \right]/d} \right\}^{-1/2}$ [4]

where d=3, the dimensionality of x_i_ and x_j_.

**1.5. Generalized Correlations Score (GCs)**

The Generalized Correlation Score (GCs) parameter has been originally introduced by Ricci et al. in the study of the conformational dynamics of the PPARγ-RXRα nuclear receptor complex ([Ricci et al., 2016](#_ENREF_25)). The GCs are computed by processing the GC matrix as follows. For each amino acid residue a GCs can be defined:

${GCs}_{i}={\sum_{j\neq i}^{N} {GC}_{ij}}$ [5]

representing a measure of both the number and the intensity of the GC coefficients displayed by each residue. To filter non-trivial correlations and eliminate the noise due to uncorrelated motions, per-residue GCs were computed considering only highly positive correlations (GC ≥ 0.60). GCs were used to detail the overall inter-domain correlations as follows: GCs were calculated for each residue *i* belonging to a specific protein domain (e.g., HNH, Rec1-3), with the residues *j* belonging to another protein domain of interest. Then, GCs were accumulated over all residues *j* of each specific Cas9 domain and normalized by the number of coupling residues *i* and *j*, which display GC ≥ 0.60. This resulted in a set of per-domain GCs, ranging from 0 (not-correlated) to 1 (correlated), measuring the strength of the overall correlation that each domain establishes with the others. The overall GCs are a measure of the most important correlated motions among protein domains taking place in the simulations, which help in identifying how specific protein regions mechanistically intervene in the overall correlation network ([Palermo et al., 2017b](#_ENREF_21); [Ricci et al., 2016](#_ENREF_25)).

**2. Supplementary Figures**

**
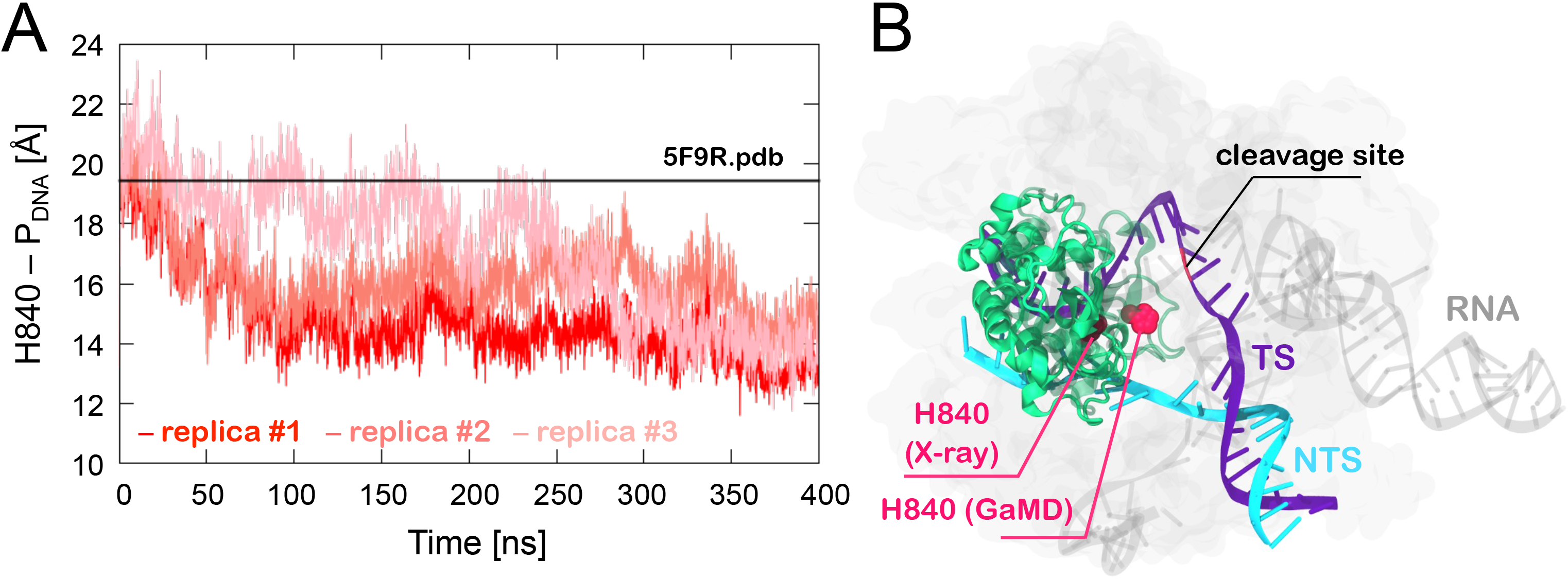
**

**Fig. S1.** (A) Time evolution of the distance between Cα@H840 and the scissile phosphate on the TS (at position G-3), along three simulation replicas of ~400 ns of the pre-activated CRISPR-Cas9 state (i.e., 5F9R.pdb) ([Jiang et al., 2016](#_ENREF_12)), performed in enhanced sampling regime using a Gaussian accelerated MD (GaMD) methodology.[^17^](#_ENREF_17)^,^[^18^](#_ENREF_18) Full details are reported in the original paper ([Palermo et al., 2017a](#_ENREF_20)). (B) A cartoon of CRISPR-Cas9 shows the repositioning of the catalytic H840 (in space filling representation, magenta) from the configuration assumed in the X-ray structure (5F95.pdb) up to the final configuration obtained from GaMD. The crystallographic pose of H840 is also shown. The Cas9 protein is shown in molecular surface, while the RNA (gray) and the DNA target (TS, violet) and non-target (NTS, cyan) strands are shown as ribbons. The HNH domain is show as cartoon in green.

**
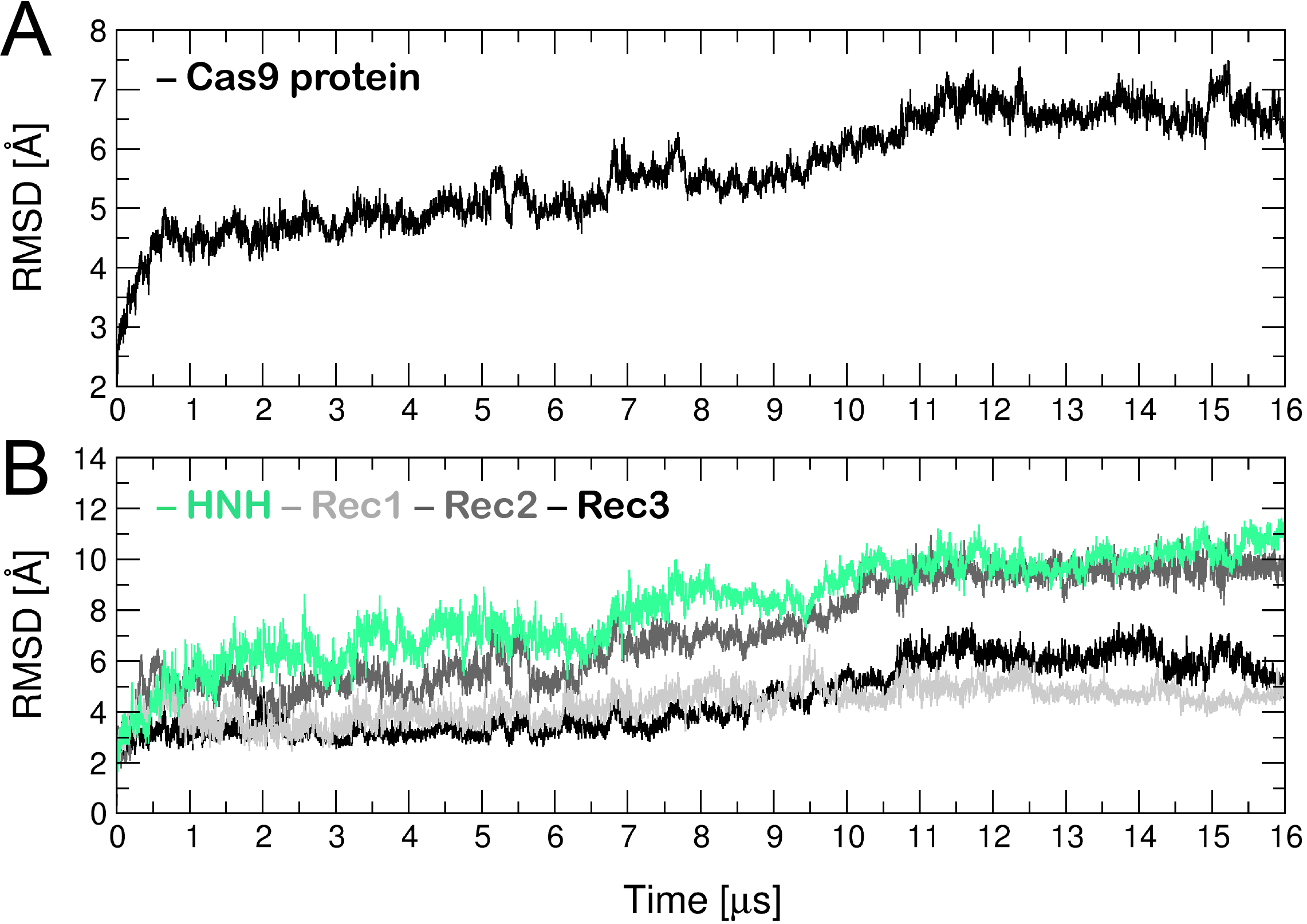
**

**Fig. S2.** (A) Time evolution along ~16 μs of MD of the Root Mean Square Deviation (RMSD) of the backbone atoms with respect to the initial MD configuration for the whole Cas9 protein (A) and for the individual HNH and REC1-3 domains (B). After ~7 μs of MD, the RMSD of the HNH domain reaches an increase at ~8 Å, as a sign of the conformational transition. At the same time, we detect a gradual increase of the RMSD for the REC2 domain.

**
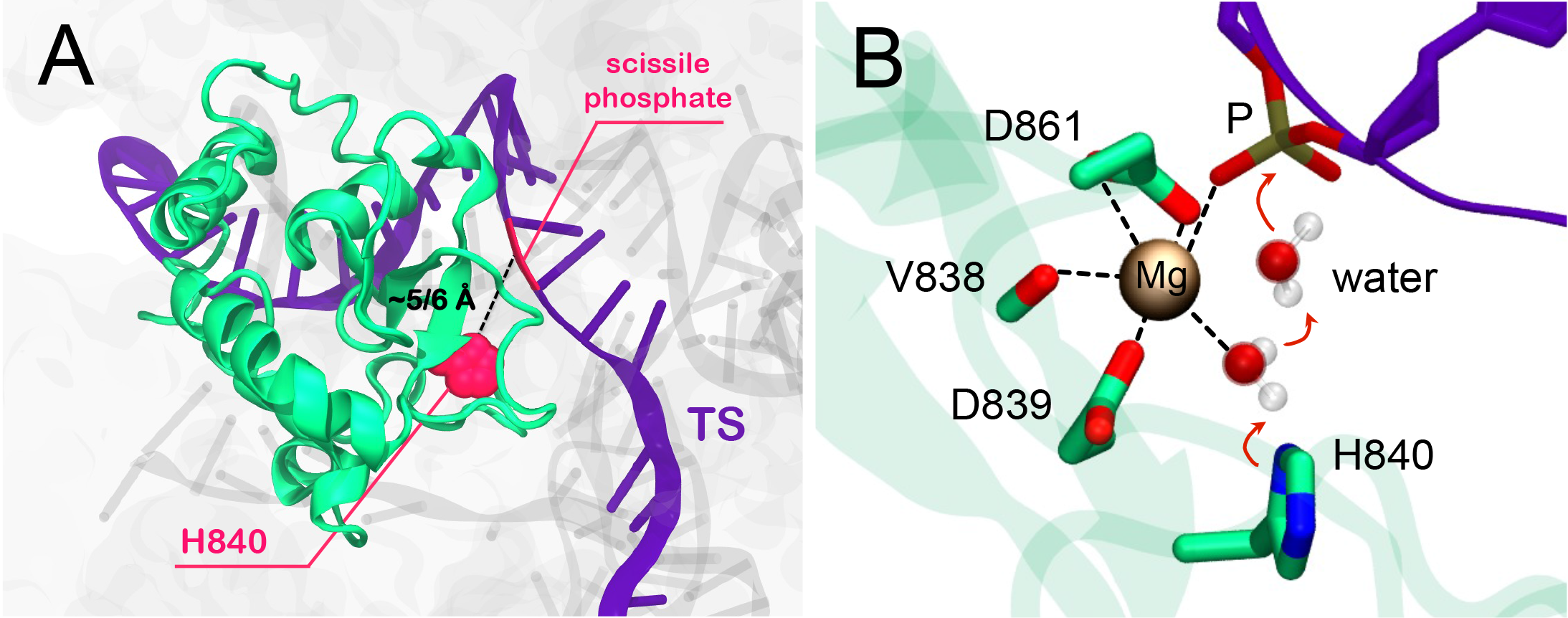
**

**Fig. S3.** (A) Representative snapshot from the last ~4 μs of MD of the CRISPR-Cas9 system, showing the HNH active site in the activated state. Cas9 is shown in molecular surface. The TS (violet), NTS (gray) and the guide RNA (gray) are shown as ribbons. The catalytic H840 (magenta) is shown in space filling representation. The imidazole side chain (and the reactive nitrogen) locates at a distance of ~5/6 Å from the scissile phosphate, allowing the entrance of water molecules for the hydrolysis. (B) Close view of the active site, showing the catalytic Mg^2+^ ion coordinated by the protein residues and by the scissile phosphate. Two water molecules locate in between the scissile phosphate and the catalytic H480 for hydrolysis, indicating a possible catalytic mechanism (shown with red arrows).


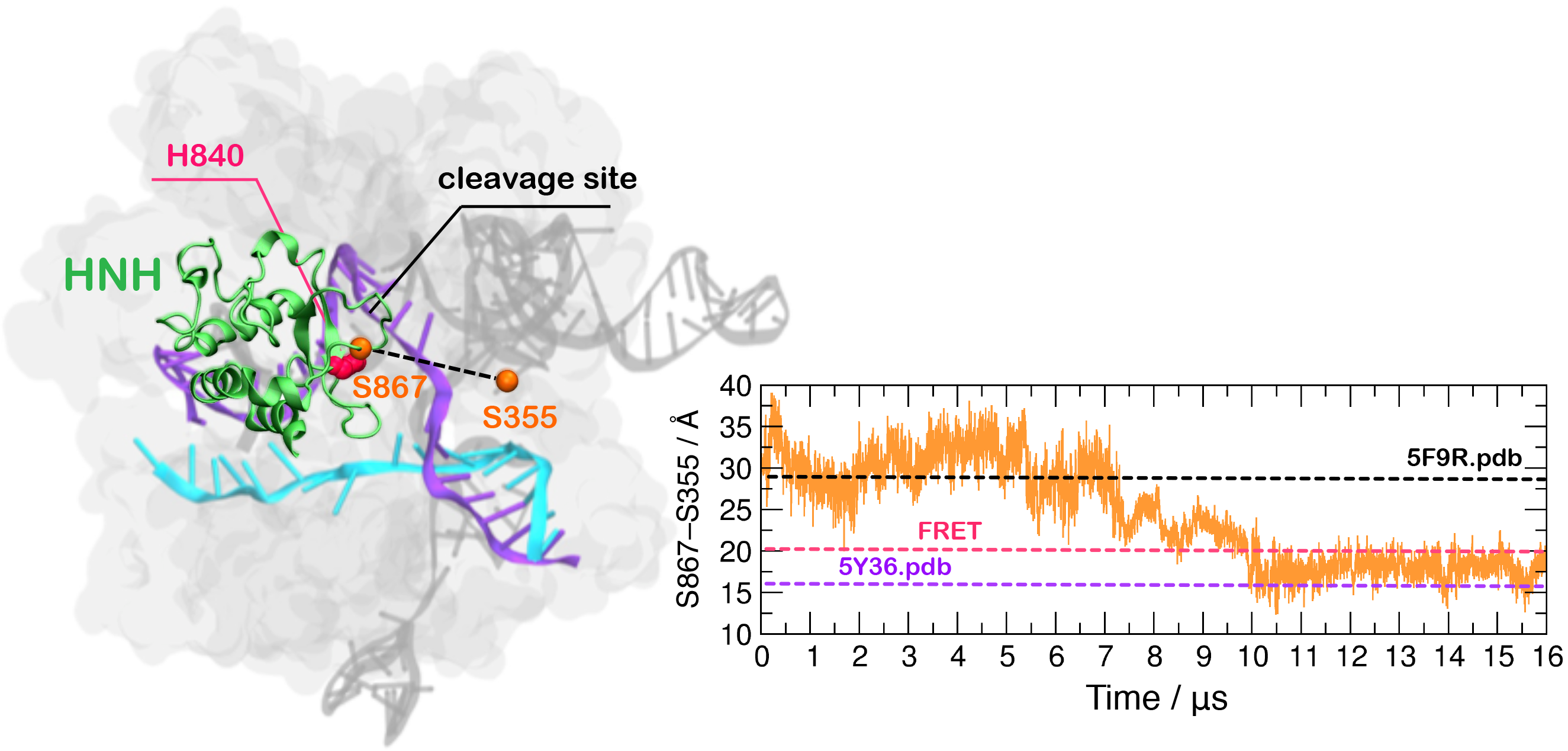


**Fig. S4.** The graph reports the time evolution along ~16 μs of MD of the distance between Cα atoms of S867 and S355, indicating the docking of the HNH domain at the cleavage site on the TS. Horizontal bars are used to indicate the value of the S867-S355 distance in the X-ray structure of the pre-activated state (5F9R.pdb ([Jiang et al., 2016](#_ENREF_12)), starting configuration for MD, black bar), in the model structure obtained via cryo-EM fitting of the EMD-8236 map (5Y36.pdb ([Huai et al., 2017](#_ENREF_10)), violet bar), and obtained via FRET and biochemical studies ([Raper et al., 2018](#_ENREF_24); [Sternberg et al., 2015](#_ENREF_31)) (magenta). During MD, the S867-S355 distance diminishes from the initial ~29.3 Å (5F9R.pdb), reaching an average of ~18 Å. This value oscillates between the value obtained via FRET experiments (~21 Å) and observed in the fitted 5Y36.pdb (16.53 Å). The picture on the left schematically shows the S867-S355 distance on the activated CRISPR-Cas9 structure. Cas9 is shown in molecular surface, with the HNH domain (green) highlighted as cartoon. The TS (violet), NTS (cyan) and the guide RNA (gray) are shown as ribbons. The catalytic H840 (magenta) is shown in space filling representation.

**
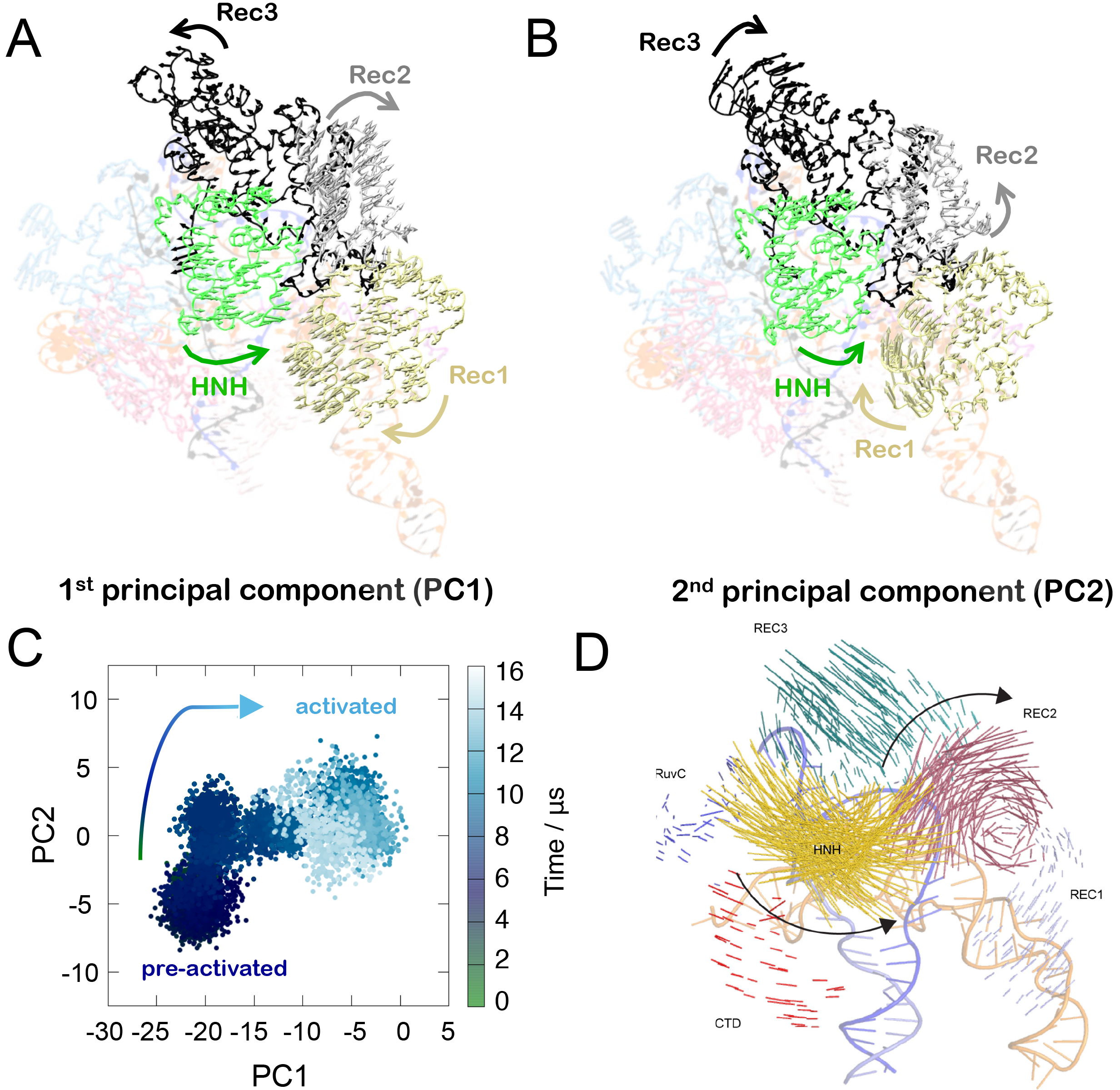
**

**Fig. S5.** (A-B) *“Essential dynamics”* derived from the first principal component PC1 (A) and from the second principal component PC2 (B), of the individual protein domains of the CRISPR-Cas9 system, as simulated using ANTON-2. The essential dynamics of the HNH domain and of the REC1-3 regions of the α-helical lobe is shown using arrows of sizes proportional to the amplitude of motions. (C) Projections of the first and second principal motions (PC1 vs PC2), derived from the simulations, characterizing the conformational space sampled by Cas9 from the pre-activated to the activated state. (D) Experimental vector map of the global Cas9 conformational changes from the RNA-bound state (4ZT0.pdb) to the DNA-bound state (5F9R.pdb). The vector map conjuncts the Cα atoms of the 4ZT0.pdb and 5F9R.pdb X-ray structures. Adapted from Chen et al. ([Chen et al., 2017](#_ENREF_8)). Remarkable agreement is observed between the first principal component (PC1, plotted on panel A) and the experimental vector map (panel D).


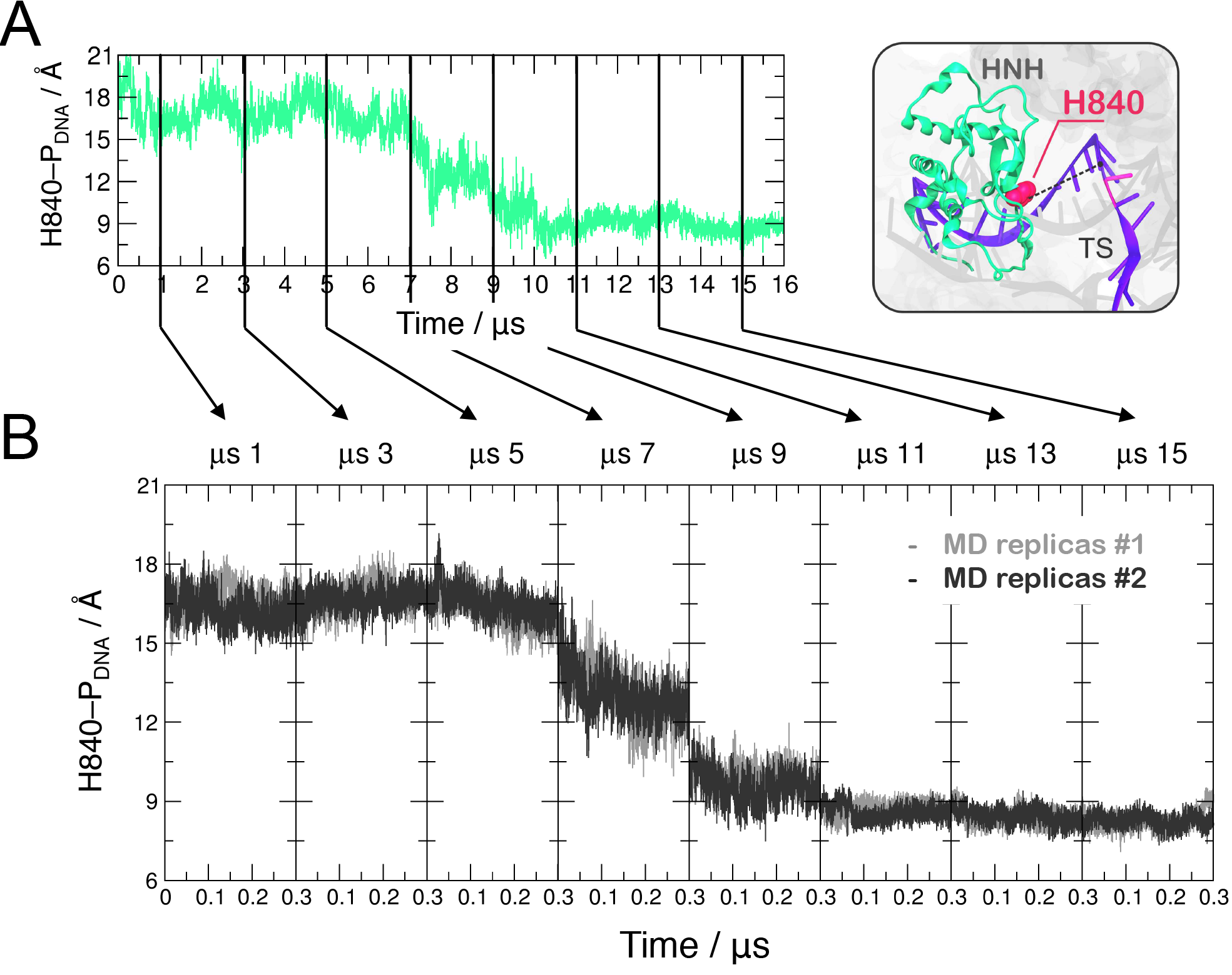


**Fig. S6.** Time evolution along ~16 μs of continuous MD of the H840–P_DNA_ distance (A) and along 300 ns of MD simulations of 8 equally distributed snapshots extracted from the continuous MD simulation at times 1, 3, 5, 7, 9, 11, 13 and 15 μs (B). For each snapshot of the CRISPR-Cas9 system, ~300 ns of independent MD simulations have been carried out in two simulations replicas (shown in black and gray, respectively). The H840–P_DNA_ distance is shown on the top left.


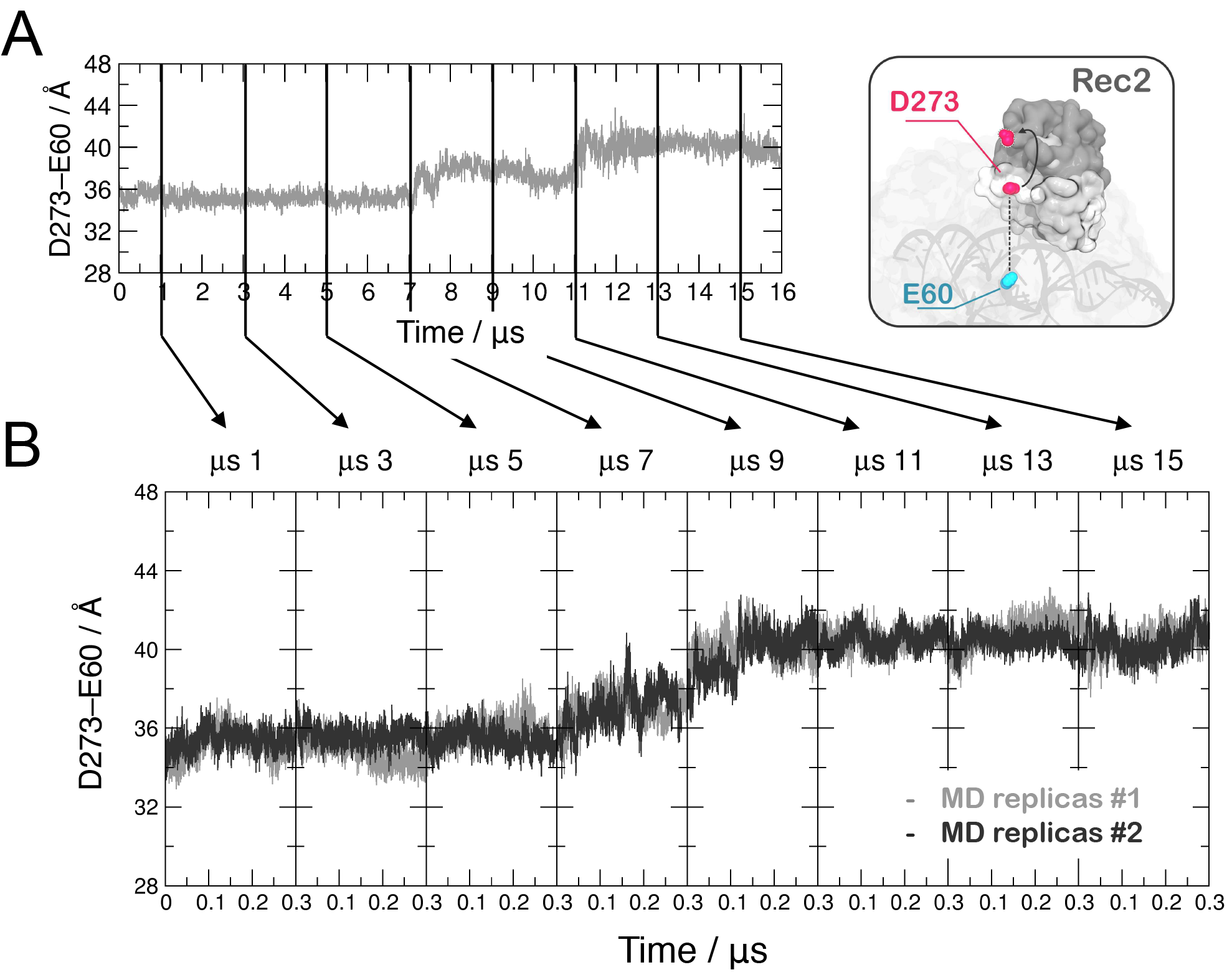


**Fig. S7.** Time evolution along ~16 μs of continuous MD of the D273–E60 distance (A) and along 300 ns of MD simulations of 8 equally distributed snapshots extracted from the continuous MD simulation at times 1, 3, 5, 7, 9, 11, 13 and 15 μs (B). For each snapshot of the CRISPR-Cas9 system, ~300 ns of independent MD simulations have been carried out in two simulations replicas (shown in black and gray, respectively). The D273–E60 distance is shown on the top left.


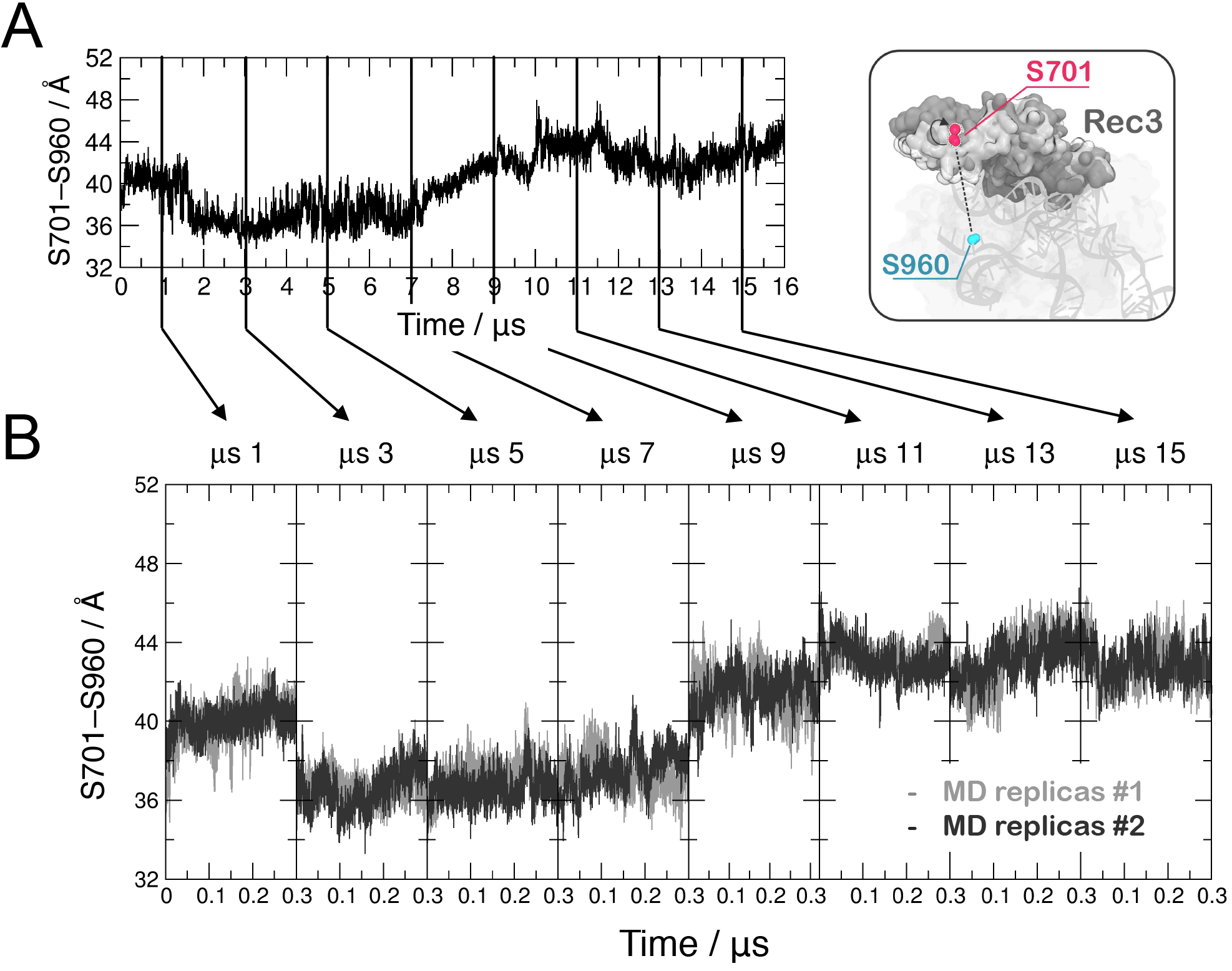


**Fig. S8.** Time evolution along ~16 μs of continuous MD of the S701–S960 distance (A) and along 300 ns of MD simulations of 8 equally distributed snapshots extracted from the continuous MD simulation at times 1, 3, 5, 7, 9, 11, 13 and 15 μs (B). For each snapshot of the CRISPR-Cas9 system, ~300 ns of independent MD simulations have been carried out in two simulations replicas (shown in black and gray, respectively). The S701–S960 distance is shown on the top left.


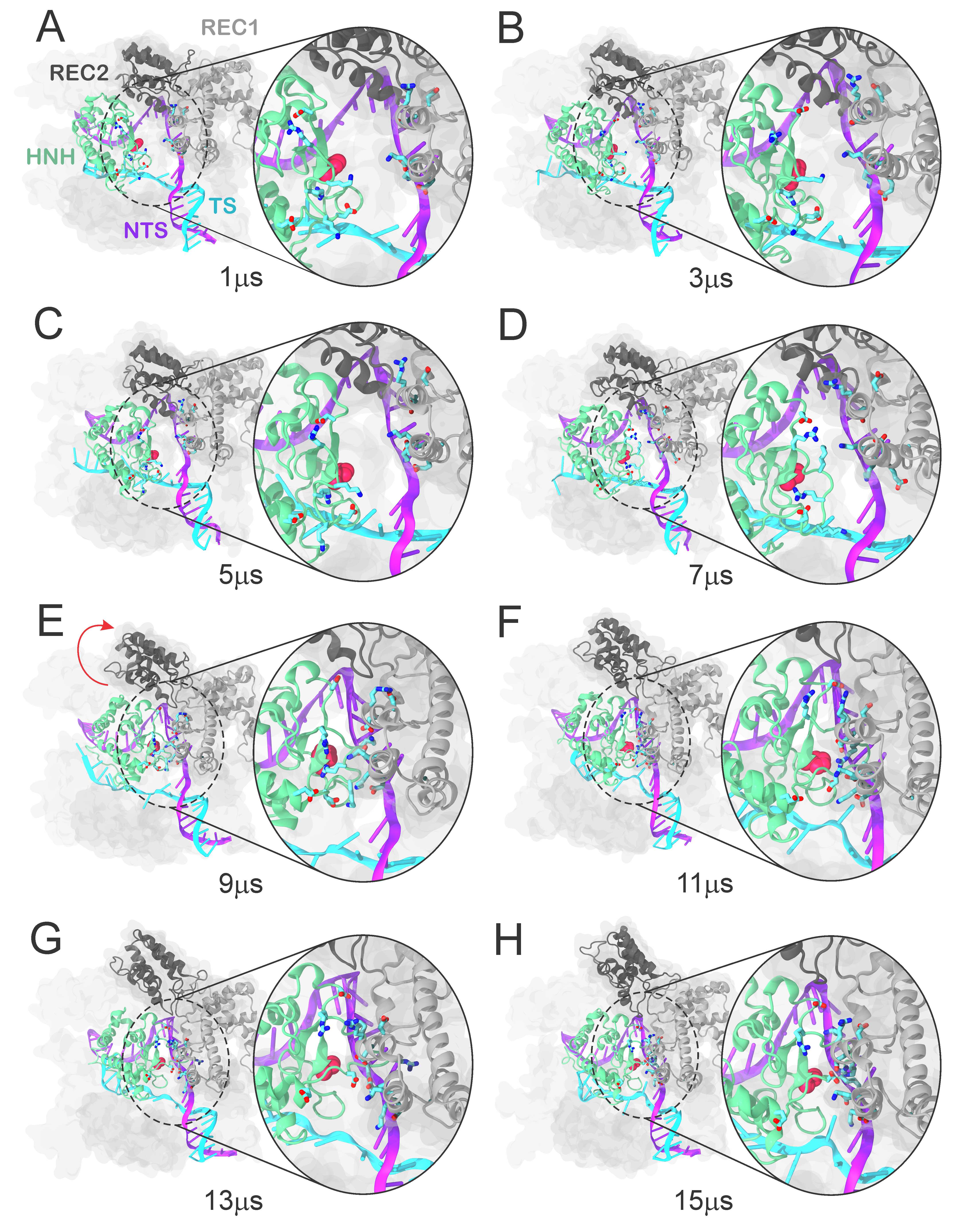


**Fig. S9.** Snapshots of CRISPR-Cas9 extracted from the continuous MD at 1, 3, 5, 7, 9, 11, 13 and 15 μs. The Cas9 protein is shown in molecular surface, highlighting the HNH (green), REC1 (light gray) and REC2 (dark gray) domains as ribbons. Residues of HNH and REC1 engaging in salt-bridges interactions are shown as sticks. The DNA TS (violet) and NTS (cyan) are shown as ribbons, while the RNA is omitted for clarity. At 7 μs (panel D), we observe the approach of the side chains of charged residues belonging to HNH and REC1. At 9 μs (panel E), the outward transition of REC2 with respect to HNH is observed and is highlighted with an arrow.

**3. Supplementary Movies**

**Movie. S1.** *“Essential dynamics”* derived from the first principal component (PC1) of the individual protein domains of the CRISPR-Cas9 system, as simulated using ANTON-2. The *“essential dynamics”* reveals large amplitude motions for HNH and the REC1-3 regions and the concerted nature of the conformational changes. The Cas9 protein is shown in molecular surface, highlighting individual protein domains with different colors. The RNA (orange), the target DNA (TS, violet) and the non-target DNA (NTS, cyan) strands are shown as ribbons.

**Movie. S2.** Conformational changes of the CRISPR-Cas9 system, observed over ~16 μs of continuous molecular dynamics (MD) simulations. During the dynamics, the catalytic HNH domain (green) spontaneously approaches the DNA target strand (violet, shown as ribbons) for cleavage. Concurrently, the recognition regions REC1-3 show significant conformational changes, with critical role for DNA cleavage. Specifically, the REC1 region intervene in “sensing” the nucleic acid binding, REC2 “regulates” the HNH conformational transition, while REC1 “locks” HNH at the cleavage site for catalysis. Molecular simulations have been performed using Anton-2, a specialized supercomputer enabling to capture micro-to-millisecond biophysical events in real time and at atomic level resolution ([www.psc.edu](http://www.psc.edu)). Music: Bach, Cello Suite No. 1, BWV 1007. K. Yamashita.

**4. Supplementary References**

AMADEI, A., LINSSEN, A. B. M. & BERENDSEN, H. J. C. (1993). Essential Dynamics of Proteins. *Proteins-Structure Function and Genetics* **17**, 412-425.

AQVIST, J. (1990). Ion-Water interaction Potentials Derived from Free Energy Perturbation Simulations. *Journal of Physical Chemistry* **94**, 8021-8024.

BANAS, P., HOLLAS, D., ZGARBOVA, M., JURECKA, P., OROZCO, M., CHEATHAM, T. E., SPONER, J. & OTYEPKA, M. (2010). Performance of Molecular Mechanics Force Fields for RNA Simulations: Stability of UUCG and GNRA Hairpins. *Journal of Chemical Theory and Computation* **6**, 3836-3849.

BERENDSEN, H. J. C., POSTMA, J. P. M., VAN GUNSTEREN, W. F., DINOLA, A. & HAAK, J. R. (1984). Molecular dynamics with coupling to an external bath. *The Journal of Chemical Physics* **81**, 3684-3690.

CASALINO, L., PALERMO, G., ABDURAKHMONOVA, N., ROTHLISBERGER, U. & MAGISTRATO, A. (2017). Development of Site-Specific Mg2+-RNA Force Field Parameters: A Dream or Reality? Guidelines from Combined Molecular Dynamics and Quantum Mechanics Simulations. *Journal of Chemical Theory and Computation* **13**, 340-352.

CASALINO, L., PALERMO, G., ROTHLISBERGER, U. & MAGISTRATO, A. (2016). Who Activates the Nucleophile in Ribozyme Catalysis? An Answer from the Splicing Mechanism of Group II Introns. *Journal of the American Chemical Society*, **138**, 10374–10377.

CASE, D. A., BETZ, R. M., BOTELLO-SMITH, W., CERUTTI, D. S., CHEATHAM, I., T. E., DARDEN, T. A., DUKE, R. E., GIESE, T. J., GOHLKE, H., GOETZ, A. W., HOMEYER, N., IZADI, S., JANOWSKI, P., KAUS, J., KOVALENKO, A., LEE, T. S., LEGRAND, S., LI, P., LIN, C., LUCHKO, T., LUO, R., MADEJ, B., MERMELSTEIN, D., MERZ, K. M., MONARD, G., NGUYEN, H., NGUYEN, H. T., OMELYAN, I., ONUFRIEV, A., ROE, D. R., ROITBERG, A., SAGUI, C., SIMMERLING, C. L., SWAILS, J., WALKER, R. C., WANG, J., WOLF, R. M., WU, X., XIAO, L., M., Y. D. & A., K. P. (2016). AMBER 2016. University of California, San Francisco.

CHEN, J. S., DAGDAS, Y. S., KLEINSTIVER, B. P., WELCH, M. M., HARRINGTON, L. B., STERNBERG, S. H., JOUNG, J. K., YILDIZ, A. & DOUDNA, J. A. (2017). Enhanced proofreading governs CRISPR-Cas9 targeting accuracy. *Nature* **550**, 407–410.

FLOYD, R. W. (1962). Algorithm-97 - Shortest Path. *Communications of the Acm* **5**, 345-345.

HUAI, C., LI, G., YAO, R. J., ZHANG, Y., CAO, M., KONG, L. L., JIA, C. Q., YUAN, H., CHEN, H. Y., LU, D. R. & HUANG, Q. (2017). Structural insights into DNA cleavage activation of CRISPR-Cas9 system. *Nature Communications* **8**, 1375.

HUMPHREY, W., DALKE, A. & SCHULTEN, K. (1996). VMD: visual molecular dynamics. *Journal of Molecular Graphics* **14**, 33-38.

JIANG, F. G., TAYLOR, D. W., CHEN, J. S., KORNFELD, J. E., ZHOU, K. H., THOMPSON, A. J., NOGALES, E. & DOUDNA, J. A. (2016). Structures of a CRISPR-Cas9 R-loop complex primed for DNA cleavage. *Science* **351**, 867-871.

JINEK, M., JIANG, F., TAYLOR, D. W., STERNBERG, S. H., KAYA, E., MA, E., ANDERS, C., HAUER, M., ZHOU, K., LIN, S., KAPLAN, M., IAVARONE, A. T., CHARPENTIER, E., NOGALES, E. & DOUDNA, J. A. (2014). Structures of Cas9 endonucleases reveal RNA-mediated conformational activation. *Science* **343**, 1247997- 1247111.

JORGENSEN, W. L., CHANDRASEKHAR, J., MADURA, J. D., IMPEY, R. W. & KLEIN, M. L. (1983). Comparison of simple potential functions for simulating liquid water. *Journal of Chemical Physics* **79**, 926-935.

LANGE, O. F. & GRUBMULLER, H. (2006). Generalized correlation for biomolecular dynamics. *Proteins-Structure Function and Bioinformatics* **62**, 1053-1061.

LE GRAND, S., GOETZ, A. W. & WALKER, R. C. (2013) SPFP: Speed without compromise—A mixed precision model for GPU accelerated molecular dynamics simulations. *Computer Physics Communications* **148**, 374–380.

LINDAHL, E., HESS, B. & VAN DER SPOEL, D. (2001). GROMACS 3.0: a package for molecular simulation and trajectory analysis. *Journal of Molecular Modeling* **7**, 306-317.

LIPPERT, R. A., PREDESCU, C., IERARDI, D. J., MACKENZIE, K. M., EASTWOOD, M. P., DROR, R. O. & SHAW, D. E. (2013). Accurate and efficient integration for molecular dynamics simulations at constant temperature and pressure. *The Journal of Chemical Physics* **139**.

PALERMO, G., CAVALLI, A., KLEIN, M. L., ALFONSO-PRIETO, M., DAL PERARO, M. & DE VIVO, M. (2015). Catalytic metal ions and enzymatic processing of DNA and RNA. *Accounts of Chemical Research* **48**, 220-228.

PALERMO, G., MIAO, Y., WALKER, R. C., JINEK, M. & MCCAMMON, J. A. (2017a). CRISPR-Cas9 conformational activation as elucidated from enhanced molecular simulations. *Proceedings of the National Academy of Sciences of the United States of America* **114**, 7260–7265.

PALERMO, G., RICCI, C. G., FERNANDO, A., RAJSHEKHAR, B., JINEK, M., RIVALTA, I., BATISTA, V. S. & MCCAMMON, J. A. (2017b). PAM-induced allostery activates CRISPR-Cas9. *Journal of the American Chemical Society* **139**, 16028–16031.

PALERMO, G., STENTA, M., CAVALLI, A., DAL PERARO, M. & DE VIVO, M. (2013). Molecular Simulations Highlight the Role of Metals in Catalysis and Inhibition of Type II Topoisomerase. *Journal of Chemical Theory and Computation* **9**, 857-862.

PEREZ, A., MARCHAN, I., SVOZIL, D., SPONER, J., CHEATHAM, T. E., 3RD, LAUGHTON, C. A. & OROZCO, M. (2007). Refinement of the AMBER force field for nucleic acids: improving the description of alpha/gamma conformers. *Biophysical Journal* **92**, 3817-3829.

RAPER, A. T., STEPHENSON, A. A. & SUO, Z. (2018). Functional Insights Revealed by the Kinetic Mechanism of CRISPR/Cas9. *Journal of the American Chemical Society* **140**, 2971−2984.

RICCI, C. G., SILVEIRA, R. L., RIVALTA, I., BATISTA, V. S. & SKAF, M. S. (2016). Allosteric Pathways in the PPAR gamma-RXR alpha nuclear receptor complex. *Scientific Reports* **6**, 19940.

RYCKAERT, J. P., CICCOTTI, G. & BERENDSEN, H. J. C. (1977). Numerical-Integration of Cartesian Equations of Motion of a System with Constraints - Molecular-Dynamics of N-Alkanes. *Journal of Computational Physics* **23**, 327-341.

SALOMON-FERRER, R., GOTZ, A. W., POOLE, D., LE GRAND, S. & WALKER, R. C. (2013). Routine Microsecond Molecular Dynamics Simulations with AMBER on GPUs. 2. Explicit Solvent Particle Mesh Ewald. *Journal of Chemical Theory and Computation* **9**, 3878-3888.

SHAN, Y. B., KLEPEIS, J. L., EASTWOOD, M. P., DROR, R. O. & SHAW, D. E. (2005). Gaussian split Ewald: A fast Ewald mesh method for molecular simulation. *Journal of Chemical Physics* **122**.

SHAW, D. E., GROSSMAN, J. P., BANK, J. A., BATSON, B., BUTTS, J. A., CHAO, J. C. & DENEROFF, M. M. (2014). Anton 2: Raising the Bar for Performance and Programmability in a Special-Purpose Molecular Dynamics Supercomputer. 41–53. IEEE, 2014. doi:10.1109/SC.2014.9.

SPONER, J., BUSSI, G., KREPL, M., BANAS, P., BOTTARO, S., CUHNA, R. A., GIL-LEY, A., PINAMONTI, G., POBLETE, S., JURECKA, P., WALTER, N. G. & OTYEPKA, M. (2018). RNA Structural Dynamics As Captured by Molecular Simulations: A Comprehensive Overview. *Chemical Reviews* **118**, 4177-4338.

STERNBERG, S. H., LAFRANCE, B., KAPLAN, M. & DOUDNA, J. A. (2015). Conformational control of DNA target cleavage by CRISPR-Cas9. *Nature* **527**, 110-113.

TUCKERMAN, M., BERNE, B. J. & MARTYNA, G. J. (1992). Reversible Multiple Time Scale Molecular-Dynamics. *Journal of Chemical Physics* **97**, 1990-2001.

TURQ, P., LANTELME, F. & FRIEDMAN, H. L. (1977). BROWNIAN DYNAMICS - ITS APPLICATION TO IONIC-SOLUTIONS. *Journal of Chemical Physics* **66**, 3039-3044.

VAN DER SPOEL, D., LINDAHL, E., HESS, B., GROENHOF, G., MARK, A. E. & BERENDSEN, H. J. C. (2005). Gromacs: Fast, Flexible, and Free. *Journal of Computational Chemistry* **26**, 1701-1718.

ZGARBOVA, M., OTYEPKA, M., SPONER, J., MLADEK, A., BANAS, P., CHEATHAM, T. E. & JURECKA, P. (2011). Refinement of the Cornell et al. Nucleic Acids Force Field Based on Reference Quantum Chemical Calculations of Glycosidic Torsion Profiles. *Journal of Chemical Theory and Computation* **7**, 2886-2902.
